# Bayesian network meta-analysis methods for combining individual participant data and aggregate data from single arm trials and randomised controlled trials

**DOI:** 10.1186/s12874-022-01657-y

**Published:** 2022-07-11

**Authors:** Janharpreet Singh, Sandro Gsteiger, Lorna Wheaton, Richard D. Riley, Keith R. Abrams, Clare L. Gillies, Sylwia Bujkiewicz

**Affiliations:** 1 Biostatistics Research Group, Department of Health Sciences, University of Leicester, Leicester, UK; 2 F. Hoffmann-La Roche Ltd, Basel, Switzerland; 3 Centre for Prognosis Research, School of Medicine, University of Keele, Staffordshire, UK; 4 Department of Statistics, University of Warwick, Coventry, UK; 5 Leicester Real World Evidence Unit, Diabetes Research Centre, University of Leicester, Leicester, UK

**Keywords:** Evidence synthesis, Network meta-analysis, Single-arm trials, Individual participant data, Arm-based methods, Bayesian hierarchical methods, Rheumatoid arthritis, Observational evidence

## Abstract

**Background:**

Increasingly in network meta-analysis (NMA), there is a need to incorporate non-randomised evidence to estimate relative treatment effects, and in particular in cases with limited randomised evidence, sometimes resulting in disconnected networks of treatments. When combining different sources of data, complex NMA methods are required to address issues associated with participant selection bias, incorporating single-arm trials (SATs), and synthesising a mixture of individual participant data (IPD) and aggregate data (AD). We develop NMA methods which synthesise data from SATs and randomised controlled trials (RCTs), using a mixture of IPD and AD, for a dichotomous outcome.

**Methods:**

We propose methods under both contrast-based (CB) and arm-based (AB) parametrisations, and extend the methods to allow for both within- and across-trial adjustments for covariate effects. To illustrate the methods, we use an applied example investigating the effectiveness of biologic disease-modifying anti-rheumatic drugs for rheumatoid arthritis (RA). We applied the methods to a dataset obtained from a literature review consisting of 14 RCTs and an artificial dataset consisting of IPD from two SATs and AD from 12 RCTs, where the artificial dataset was created by removing the control arms from the only two trials assessing tocilizumab in the original dataset.

**Results:**

Without adjustment for covariates, the CB method with independent baseline response parameters (*CBunadjInd*) underestimated the effectiveness of tocilizumab when applied to the artificial dataset compared to the original dataset, albeit with significant overlap in posterior distributions for treatment effect parameters. The CB method with exchangeable baseline response parameters produced effectiveness estimates in agreement with *CBunadjInd*, when the predicted baseline response estimates were similar to the observed baseline response. After adjustment for RA duration, there was a reduction in across-trial heterogeneity in baseline response but little change in treatment effect estimates.

**Conclusions:**

Our findings suggest incorporating SATs in NMA may be useful in some situations where a treatment is disconnected from a network of comparator treatments, due to a lack of comparative evidence, to estimate relative treatment effects. The reliability of effect estimates based on data from SATs may depend on adjustment for covariate effects, although further research is required to understand this in more detail.

**Supplementary Information:**

The online version contains supplementary material available at (10.1186/s12874-022-01657-y).

## Background

Traditionally, meta-analysis of randomised controlled trials (RCTs) has been used to quantitatively pool published evidence on the effectiveness of a treatment, or a network of treatments through network meta-analysis (NMA), across trials with similar characteristics (e.g. patient population, trial design and conduct) [1–3]. In the published literature, results from a trial are usually reported as aggregate data (AD) summarising the average treatment effect. Synthesising evidence from RCTs has been considered a gold standard as randomised treatment allocation minimises the risk of confounding in treatment effect estimates.

In circumstances where randomised evidence is limited, non-randomised evidence, such as data from single-arm trials (SATs), may have to be considered in determining treatment effectiveness [4]. Such cases are becoming increasingly common in health technology assessment (HTA), as new technologies receive accelerated approval from regulatory agencies based on SATs leading to disconnected networks of treatments [5]. This creates an issue for reimbursement decision-making by HTA bodies where the interest is in unbiased estimates of relative treatment effects. Consequently, there is a need for synthesis methods which combine randomised and non-randomised evidence, whilst addressing issues associated with the latter, such as susceptibility to confounding and the lack of evidence from a comparator arm in the case of SATs [6].

For a particular trial, individual participant data (IPD) may be available recording the effect of the treatment on outcomes of interest, as well as a set of covariates, for each participant. Meta-analysis incorporating IPD has been shown to overcome issues associated with detecting treatment-covariate interactions, and prognostic effects, compared to a meta-regression of AD only [7, 8]. Moreover, it accounts for variability in covariate values across participants within a trial, allowing within- and across-trial interactions to be estimated separately which mitigates the risk of aggregation bias [9, 10]. However, the availability of IPD is often subject to a data-sharing agreement with the trial sponsor which often involves a lengthy, complex process due to regulatory considerations (e.g. data protection and privacy). Furthermore, access to IPD may be particularly challenging when data are required from a number of trials, and for trials assessing different treatments developed by different manufacturers. Consequently, IPD are unlikely to be available for all trials in the synthesis and methods have been developed which combine a mixture of IPD and AD [11–14]. Population-adjustment methods have been developed to estimate a relative treatment effect for the specific situation where IPD are available for a trial assessing a particular treatment and only AD are available for another trial assessing a comparator, which is a common occurrence in HTA [15]. Such methods make the assumption that the treatment effect is equivalent in the two study populations at each level of a set of effect modifiers [16]. More recently, multilevel network meta-regression methods have been proposed which extend the population-adjustment methods beyond the two-study case, and also avoid the issue of aggregation bias by using IPD to inform the covariate distributions in the AD model [17]. Similarly, methods have been proposed which use IPD to estimate regression coefficients for effect modifiers, and synthesise IPD and AD via either a hierarchical model or by constructing empirical prior distributions [18].

Extensions to meta-analysis, which allow the synthesis of SATs and RCTs, have been proposed which assume that baseline response parameters are exchangeable (rather than independent) across trials [19]. This approach has been developed to a NMA context using a mixture of IPD and AD and a contrast-based approach to NMA [20]. Arm-based NMA methods, which parametrise absolute treatment effects across trial arms, also allow SATs to be incorporated into a synthesis of RCTs in a pairwise meta-analysis [21] and a NMA context [22, 23]. Under a Bayesian framework, these methods allow data from SATs to provide information on the pooled treatment effect and between-study heterogeneity parameters via a prior distribution, and can be down-weighted according to commensurability with RCT data [24]. These methods have been criticised for compromising randomised treatment allocation and introducing bias in treatment effect estimates from RCTs [25, 26]. However, they may be helpful to include when the only evidence available for a treatment is from SATs, and the increase in risk of bias associated with the exchangeability assumption may have little impact in practice [27, 28]. Alternative methods seek to match similar trial arms based on reported characteristics and either entering the matched pairs into the NMA directly or by plugging-in the baseline response estimate from a matched trial to estimate a relative treatment effect from SATs [29, 30].

In this paper, we develop methods to synthesise SATs and two-arm RCTs, using a mixture of IPD and AD, for a dichotomous outcome. We consider methods with and without adjustment for a covariate effect. We build on contrast- and arm-based NMA methods [22, 23] to incorporate SATs, and use shared-parameters to synthesise IPD and AD [13, 14]. We apply the developed methods using mixture of IPD and AD from RCTs assessing the effectiveness of biologic disease-modifying anti-rheumatic drugs (bDMARDs) as treatments for rheumatoid arthritis (RA), as an applied example. We describe the IPD and AD datasets used in the applied example in “Applied example: assessing biologics for treating rheumatoid arthritis (RA)” section, before detailing the methods for a Bayesian implementation in “Methods” section, and reporting the results from the application in “Results” section. In “Discussion” section, we discuss issues related to the use of the methods and suggest how these can be investigated further.

## Applied example: assessing biologics for treating rheumatoid arthritis (RA)

In this paper, we consider a case-study in rheumatoid arthritis (RA); a chronic auto-immune condition causing joint inflammation in (mostly) elderly patients. The American College of Rheumatology (ACR) response criteria provide a measure of patient response to treatment, with an ACR20 outcome representing a 20% improvement in RA symptoms as defined by the criteria [31]. A number of bDMARDs (hereafter referred to as biologics) have been developed to offer a choice of treatment strategies in managing the disease [32].

Table 1 summarises the original dataset used in the applied example. Data were available from 14 RCTs, each assessing the effectiveness of a first-line biologic versus placebo as treatment for RA, with 5,821 participants in total. In 9 trials, a biologic was allocated to multiple trial arms (ranging from two to four arms) in order to assess different dosage regimens. For this applied example, these data were aggregated and considered as from a single trial arm. There was not significant variability in dosage regimens across trials assessing a particular biologic. All trial arms consisted of participants receiving methotrexate as background therapy. IPD were available for two trials of tocilizumab [33, 34], whilst only AD were available for the remaining trials [35–49]. An ACR20 response was considered as an outcome to measure treatment effectiveness, with IPD providing responder status for each participant and AD providing the number of participants achieving a response in each trial arm. We considered RA duration (in years) as a covariate in our models, as there was evidence of an association between baseline response and RA duration present in the exploratory analysis of the IPD. Table 1 lists the mean RA duration at baseline in each trial arm.

**Table 1:** Summary (arm-level) data from RCTs assessing biologic therapies as treatments for RA, (N - number of participants, ACR20 (%) - percent of ACR20 responders)

Trial	Lead author	Year	Data type	Intervention	N	ACR20 (%)	Mean RA duration (years)
1	Genovese	2008	IPD	Tocilizumab	803	60.8	9.8
1	Genovese	2008	IPD	Placebo	413	24.5	9.8
2	Smolen	2008	IPD	Tocilizumab	418	53.1	7.4
2	Smolen	2008	IPD	Placebo	204	26.5	7.8
3	Maini	1999	AD	Infliximab	340	57.4	8.3
3	Maini	1999	AD	Placebo	88	22.7	8.9
4	Weinblatt	2003	AD	Adalimumab	209	60.3	12.7
4	Weinblatt	2003	AD	Placebo	62	14.5	11.1
5	Keystone	2004	AD	Adalimumab	419	62.1	11.0
5	Keystone	2004	AD	Placebo	200	29.5	10.9
6	Chen	2008	AD	Adalimumab	35	54.3	6.2
6	Chen	2008	AD	Placebo	12	33.3	8.3
7	Kay	2008	AD	Golimumab	137	61.3	7.9
7	Kay	2008	AD	Placebo	35	37.1	5.6
8	Keystone	2009	AD	Golimumab	178	59.6	5.6
8	Keystone	2009	AD	Placebo	133	27.8	6.5
9	Kremer	2010	AD	Golimumab	257	43.6	8.8
9	Kremer	2010	AD	Placebo	129	24.8	7.4
10	Schiff	2011	AD	Infliximab	165	59.4	7.3
10	Schiff	2011	AD	Placebo	110	41.8	8.4
11	Emery	2013	AD	Golimumab	318	61.6	3.5
11	Emery	2013	AD	Placebo	160	49.4	2.9
12	Kim	2013	AD	Infliximab	71	50.7	9.8
12	Kim	2013	AD	Placebo	72	30.6	7.4
13	Tanaka	2013	AD	Golimumab	173	72.8	8.4
13	Tanaka	2013	AD	Placebo	88	33.0	8.7
14	Weinblatt	2013	AD	Golimumab	395	58.5	6.9
14	Weinblatt	2013	AD	Placebo	197	24.9	7.0

**Fig. 1:**
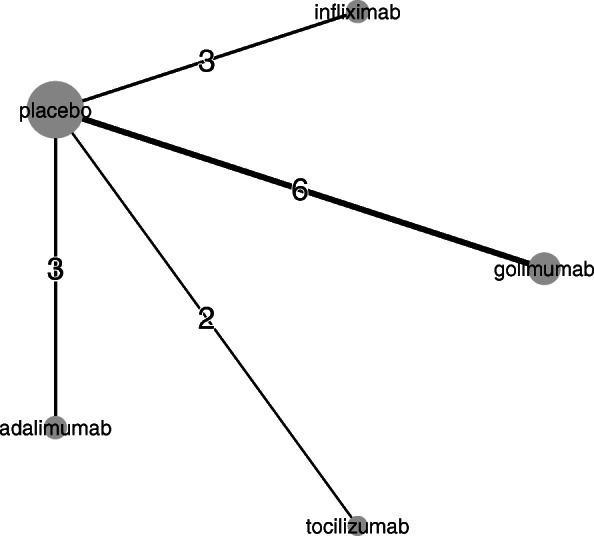
Network diagram representing number of RCTs assessing biologics as treatments for RA, IPD were available for the two trials assessing tocilizumab

Figure 1 illustrates the network structure in terms of the evidence available for each treatment comparison. The network consists of trials assessing four biologics; infliximab, tocilizumab, golimumab, and adalimumab. Each trial compared a biologic therapy versus placebo, and there were no trials comparing biologics head-to-head.

In this paper, we consider methods which can be applied to synthesise data from SATs and RCTs, in a NMA context. For the applied example, an artificial dataset was created by removing the placebo arms from the two tocilizumab trials. As a result, the artificial dataset included two SATs assessing tocilizumab. The aim of the synthesis was to compare the effectiveness of tocilizumab with infliximab, golimumab, and adalimumab, in terms of the ACR20 outcome.

## Methods

In this section, we describe Bayesian NMA methods used to synthesise data from SATs and two-arm RCTs, under both contrast- and arm-based model parametrisations. We focus on one-stage methods which synthesise IPD and AD in a single model, as opposed to two-stage methods where IPD are first aggregated and then synthesised with AD. We assume data are available as IPD from *N*^*IPD*^ SATs followed by AD from *N*^*AD*^ two-arm RCTs, and let *j*=1,...,*N*^*IPD*^,...,*N*^*IPD*^+*N*^*AD*^ index both trial sets. We start by describing the likelihood functions used to model a dichotomous outcome using IPD and AD, then describe methods under a contrast-based parametrisation, followed by methods under an arm-based parametrisation. Under each parametrisation, we describe methods without adjustment for covariates and methods which include a covariate to adjust for its effect.

### Likelihood models for a dichotomous outcome

Let *Y*_*ijk*_ represent a dichotomous outcome indicating responder status (i.e. responder 1, non-responder 0) for participant *i* in trial *j* assigned to treatment *k*. Then, for trials from which IPD are available, we assume that the outcome *Y*_*ijk*_ is a Bernoulli random variable,

1 $$\begin{array}{*{20}l}  Y_{ijk} \sim Bern(p_{ijk}), \quad {j = 1,..., N^{IPD};} \end{array} $$

where *p*_*ijk*_ represents the probability of response for each participant.

Similarly, let *n*_*jk*_ and *r*_*jk*_ represent the numbers of participants and responders, respectively, in trial *j* allocated treatment *k*. Then, for trials from which only AD are available, the number of responders in each arm *r*_*jk*_ is assumed to follow a Binomial distribution,

2 $$\begin{array}{*{20}l}  {}r_{jk} \sim Bin(n_{jk}, p_{jk}), \quad {j = N^{IPD} + 1,..., N^{IPD} + N^{AD};} \end{array} $$

where *p*_*jk*_ is the probability of response in each trial arm. For both data-types, we employ the logit link function to transform response probabilities onto the linear predictor scale on which treatment effects can be assumed to be linearly additive.

The number of responders in each arm *r*_*jk*_ is the sum over the participant-level Bernoulli outcomes *Y*_*ijk*_, which are assumed to be identically-distributed. If there are unobserved participant-level covariates which influence the individual response probabilities *p*_*ijk*_, then the corresponding outcomes *Y*_*ijk*_ are unlikely to be identically-distributed. Thus, the AD likelihood in Eq. (2) is the correct aggregate version of the IPD likelihood in Eq. (1) only if there are no important unobserved participant-level covariates.

### Contrast-based (CB) methods

Here, we describe methods under a contrast-based parametrisation where treatment effects are expressed relative to a baseline response (i.e. the outcome observed in the control arm). We denote *B* as index for the baseline treatment in each trial.

#### Methods without covariates

We start by describing methods which only model the response data and do not include data on covariates, as an intermediate step so that it is clear how these methods can be extended to incorporate covariate adjust- ment in “Methods with covariate-adjustment” section. The IPD and AD models are equivalent, but are described separately to clarify how shared-parameters allow both datasets to contribute to the estimation of the treatment effect.

For IPD, the model is given by,

3 $$\begin{array}{*{20}l}  logit(p_{ijk}) = \phi_{j} + \delta_{jBk} \times I_{ijk}, \quad {j = 1,..., N^{IPD};} \end{array} $$

where *ϕ*_*j*_ represents the log odds of a response on the baseline treatment *B* in trial *j*, *δ*_*jBk*_ is the log odds ratio of effect on treatment *k* relative to *B*, and *I*_*ijk*_ is a participant-level treatment indicator variable (i.e. treatment 1, baseline 0).

Similarly, for AD, the model can be written as,

4 $$ \begin{aligned} logit(p_{jk}) &= \phi_{j} + \delta_{jBk} \times I_{jk},\\ j &= N^{IPD} + 1,..., N^{IPD} + N^{AD}; \end{aligned}  $$

where *ϕ*_*j*_ and *δ*_*jBk*_ have the same interpretation as for the IPD model (3), and *I*_*jk*_ is an arm-level treatment indicator variable.

The treatment effects *δ*_*jBk*_ are assumed to be exchangeable across trials comparing treatment *k* with *B*,

5 $$\begin{array}{*{20}l} \delta_{jBk} \sim N\left(d_{Bk}, \sigma^{2}\right), {\quad j = 1,..., N^{IPD} + N^{AD};} \end{array} $$

with *d*_*Bk*_ representing the pooled log odds ratio. The between-study heterogeneity in the treatment effects is quantified by the standard deviation parameter *σ*. Here, *σ* is assumed to be common across treatment comparisons as we consider methods for application in cases where data are limited.

The pooled effects *d*_*Bk*_ can be represented in terms of basic parameters by assuming consistency within the network *d*_*Bk*_=*d*_1*k*_−*d*_1*B*_ (*d*_11_=0). Thus, effect estimates can be obtained indirectly for treatment comparisons for which there are no trials providing a head-to-head estimate. We place a non-informative Normal prior distribution on each of the basic parameters *d*_1*k*_∼*N*(0,10^2^) and the baseline response parameters *ϕ*_*j*_∼*N*(0,10^2^). Hong et al. place a non-informative Uniform prior distribution for the between-study heterogeneity parameter, *σ*∼*U*(0,10). However, we note that this prior distribution may be weakly-informative in a synthesis with few trials [50] and *σ*∼*U*(0,2) has been recommended as a more appropriate prior distribution [51] which we use here (The results of a sensitivity analysis using *σ*∼*U*(0,10) are reported in Appendix E for comparison).

Traditionally, NMA methods have been applied to synthesise data from RCTs which assume independent baseline response parameters *ϕ*_*j*_ [3]. This accounts for differences in prognostic factors between trials, and ensures that the impact of randomised treatment allocation is reflected in the treatment effect estimates. However, such an approach is restricted to trials with two or more arms and the interest here is to incorporate data from SATs into the synthesis. Therefore, we assume exchangeable baseline response parameters as in [19],

6 $$\begin{array}{*{20}l} \phi_{j} \sim N\left(m_{\phi}, \sigma_{\phi}^{2}\right), \quad {j = 1,..., N^{IPD} + N^{AD};} \end{array} $$

where *m*_*ϕ*_ represents the mean baseline response, and *σ*_*ϕ*_ quantifies between-study heterogeneity in the baseline response. The benefit of this assumption is that it allows a baseline response to be predicted, and a treatment effect to be estimated, for each SAT in the synthesis so that pooled estimates are based on all of the available evidence. The cost of this assumption is that randomisation is compromised for RCTs in the synthesis, which will bias treatment effect estimates where there is significant between-study heterogeneity (which may be due to differences in prognostic factors) in baseline response estimates. We specify a non-informative Normal prior distribution for the mean baseline response parameter *m*_*ϕ*_∼*N*(0,10^2^), and a non-informative Uniform prior distribution for the between-study heterogeneity parameter *σ*_*ϕ*_∼*U*(0,2). We label the methods with independent and exchangeable baseline response parameters by *CBunadjInd* and *CBunadjEx*, respectively.

Alternatively, the *CBunadjInd* method can be implemented in two stages to predict baseline response estimates for SATs in the synthesis, whilst maintaining the effects of randomisation and limiting bias in treatment effect estimates for RCTs. In the first stage, the *CBunadjEx* method is applied to the data from the set of RCTs in the synthesis. The posterior estimates for the mean and standard deviation parameters (i.e. $\hat {m}_{\phi }$ and $\hat {\sigma }_{\phi }$), describing the distribution of the baseline response across RCTs, are recorded. In the second stage, the *CBunadjInd* method is applied to synthesise SATs and RCTs, where an informative Normal prior distribution is placed on the baseline response parameters corresponding to SATs (i.e. $\phi _{j} \sim N\left (\hat {m}_{\phi }, \hat {\sigma }_{\phi }^{2}\right)$). This prior distribution is informed by the posterior estimates recorded from the first stage of the method, which is equivalent to using the posterior predictive distribution of the baseline response across RCTs. The posterior predictive distribution, as opposed to the posterior distribution, is recommended by Dias et al. to account for between-study heterogeneity [52]. Ultimately, pooled treatment effect estimates, and inference, are based only on the second stage of the method. Thus, SATs are incorporated into the synthesis and contribute to the pooled treatment effect estimates, whilst the assumption of independent baseline response parameters ensures randomisation in RCTs is not compromised.

#### Methods with covariate-adjustment

##### Study-level adjustment only

Here, we consider methods which include a covariate to adjust the baseline response. We begin by extending the *CBunadjEx* method to adjust for a covariate at the trial-level, and let $\bar {x}_{j}$ represent the mean covariate value summarising participants in trial *j*. We use the superscripts *A* and *W* to denote across- and within-trial effects, respectively. For IPD, the extended model is given by,

7 $$\begin{array}{*{20}l}  {}logit(p_{ijk}) = \phi_{j} + \alpha^{A} \times \bar{x}_{j} + \delta_{jBk} \times I_{ijk}, \quad {j = 1,..., N^{IPD};} \end{array} $$

where *ϕ*_*j*_ represents the log odds of response on baseline treatment *B* when $\bar {x}_{j}$ is equal to zero, and *α*^*A*^ is the change in the log odds for a unit increase in $\bar {x}_{j}$. Thus, *α*^*A*^ represents a trial-level covariate effect, accounting for the association between the baseline response *ϕ*_*j*_ and the mean covariate $\bar {x}_{j}$, across trials. Here, there is no adjustment for the interaction between the relative treatment effect and the mean covariate, and so *δ*_*jBk*_ represents a marginal treatment effect.

Similarly, for AD, the extended model can be described by,

8 $$ {}\begin{aligned} logit(p_{jk}) &= \phi_{j} + \alpha^{A} \times \bar{x}_{j} + \delta_{jBk} \times I_{jk},\\ j &= N^{IPD} + 1,..., N^{IPD} + N^{AD}; \end{aligned}  $$

where *ϕ*_*j*_ and *α*^*A*^ have the same interpretation as for the IPD model (7). A non-informative Normal prior distribution is recommended for the across-trial covariate effect parameter *α*^*A*^∼*N*(0,10^2^), and we label this method *CBadjEx*.

##### Participant-level and study-level adjustment

In this paper, we consider methods to synthesise a mixture of IPD and AD. IPD allows participant-level and trial-level covariate effects to be estimated separately, avoiding aggregation bias which may occur when the assumption that these effects are equivalent does not hold [8]. We extend the *CBadjEx* method in Eq. (7) to adjust for the covariate both at the participant-level and the trial-level. We let *x*_*ijk*_ represent the covariate value for participant *i* in trial *j* allocated treatment *k*. For IPD, the extended model is given by,

9 $$ {} \begin{aligned} logit(p_{ijk}) &= \phi_{j} + \alpha^{A} \times \bar{x}_{j} + \alpha^{W} \times \left(x_{ijk} - \bar{x}_{j} \right) + \delta_{jBk} \times I_{ijk},\\ j &= 1,..., N^{IPD}; \end{aligned}  $$

where *α*^*W*^ is the change in the log odds of a response on baseline treatment *B* for a unit increase in $\left (x_{ijk} - \bar {x}_{j} \right)$. Thus, *α*^*W*^ represents a common (participant-level) covariate effect within a trial. A non-informative Normal prior distribution is recommended for within-trial covariate effect parameter *α*^*W*^∼*N*(0,10^2^), and we label this method *CBadjEbEx* (where *Eb* denotes that this method accounts for ecological aggregation bias - the difference between the across- and within-trial covariate effects).

### Arm-based (AB) methods

Hong et al. have proposed NMA models under an arm-based parametrisation, where treatment effects are expressed as the absolute effects across trial arms, using either IPD [22] or AD [23]. Here, we extend their models to synthesise a mixture of IPD and AD.

#### Methods without covariates

We begin by describing models with outcome data only. For IPD, the model is given by,

10 $$\begin{array}{*{20}l}  logit(p_{ijk}) = \theta_{k} + \nu_{jk}, \quad {j = 1,..., N^{IPD};} \end{array} $$

where *θ*_*k*_ represents the pooled log odds of response on treatment *k*, and *ν*_*jk*_ are error terms (i.e. the difference between the log odds of response on treatment *k* in trial *j* and the pooled log odds of response on treatment *k*). A non-informative Normal prior distribution is suggested for each pooled response parameter *θ*_*k*_∼*N*(0,10^2^).

Similarly, for AD, the model is described by,

11 $$\begin{array}{*{20}l} {}logit(p_{jk}) = \theta_{k} + \nu_{jk}, \quad {j = N^{IPD} + 1,..., N^{IPD} + N^{AD};} \end{array} $$

where *θ*_*k*_ and *ν*_*jk*_ have the same interpretation as for the IPD model (10).

The error terms *ν*_*jk*_ are assumed to be exchangeable across trials,

12 $$\begin{array}{*{20}l} \nu_{jk} \sim N\left(0, \tau^{2}\right), \quad {j = 1,..., N^{IPD} + N^{AD};} \end{array} $$

where *τ* quantifies the between-study heterogeneity and is assumed to be common. A non-informative Uniform prior distribution is recommended for the between-study heterogeneity parameter *τ*∼*U*(0,2).

The application in this paper considers a synthesis of SATs and two-arm RCTs, and we make an additional assumption regarding the heterogeneity observed in the latter. For each two-arm RCT, we let *k*_1_ and *k*_2_ denote the treatment assigned in arms 1 and 2, respectively. Then, the error terms are assumed to follow a bivariate distribution,

13 $$ \begin{aligned} \left(\begin{array}{l} \nu_{jk_{1}} \\ \nu_{jk_{2}} \end{array}\right) &\sim N \left(\left(\begin{array}{l} 0 \\ 0 \end{array}\right), \boldsymbol{\Sigma}_{k_{1} k_{2}} = \left(\begin{array}{ll} \tau^{2} & \rho \tau^{2} \\ \rho \tau^{2} & \tau^{2} \end{array}\right) \right),\\ j &= N^{IPD} + 1,..., N^{IPD} + N^{AD}; \end{aligned}  $$

with covariance matrix $\boldsymbol {\Sigma }_{k_{1} k_{2}}$. The responses observed in arms from RCTs are likely to be similar (compared to two SATs), due to the balance in prognostic factors achieved by randomised treatment allocation [53]. The similarity is represented by a common distribution, which is a bivariate distribution in the case of data from two-arm RCTs, accounting for the correlation between treatment arms. A non-informative Uniform prior distribution is suggested for the correlation parameter in the covariance matrix *ρ*∼*U*(−1,1). We label this method as *ABunadj*.

#### Methods with covariate-adjustment

##### Study-level adjustment only

Here, we extend the *ABunadj* method to allow adjustment for a covariate. We begin by adjusting the absolute treatment effect in each arm for the average value of the covariate $\bar {x}_{jk}$ across participants in trial *j* and treatment arm *k*. For IPD, the extended model is given by,

14 $$\begin{array}{*{20}l}  {}logit(p_{ijk}) = \theta_{k} + \nu_{jk} + \beta^{A} \times \bar{x}_{jk}, \quad {j = 1,..., N^{IPD};} \end{array} $$

where *θ*_*k*_ represents the pooled log odds of response to treatment *k* when $\bar {x}_{jk}$ is equal to zero, and *β*^*A*^ is the change in the pooled log odds for a unit increase in $\bar {x}_{jk}$. Thus, *β*^*A*^ represents the covariate effect across trial arms. Under the arm-based parametrisation, we follow the approach by Hong et al. and adjust for the arm-level mean covariate to account for differences between arms within each trial, in addition to differences between trials [22]. In some cases, data on the arm-level mean covariate $\bar {x}_{jk}$ may not be available from the published trial report, in which case adjustment would be restricted to the reported mean trial-level covariate $\bar {x}_{j}$.

Similarly, for AD, the extended model can be written as,

15 $$ \begin{aligned} logit(p_{jk}) &= \theta_{k} + \nu_{jk} + \beta^{A} \times \bar{x}_{jk},\\ j &= N^{IPD} + 1,..., N^{IPD} + N^{AD}; \end{aligned}  $$

where *θ*_*k*_ and *β*^*A*^ have the same interpretation as for the IPD model (14). We suggest a non-informative Normal prior distribution for the arm-level covariate effect parameter *β*^*A*^∼*N*(0,10^2^), and we label this method *ABadj*.

##### Participant-level and study-level adjustment

In a similar way to that described for the contrast-based methods, the covariate effect at the participant-level may differ from the effect at the arm-level. To avoid the aggregation bias which is introduced as a result of this difference, we extend the *ABadj* method in Eq. (14) to separate the across- and within-trial covariate effects. For IPD the extended model is given by,

16 $$ {}\begin{aligned} logit(p_{ijk}) &= \theta_{k} + \nu_{jk} + \beta^{A} \times \bar{x}_{jk} + \beta^{W} \times \left(x_{ijk} - \bar{x}_{jk} \right),\\ j &= 1,..., N^{IPD}; \end{aligned}  $$

where *β*^*W*^ represents the change in the pooled log odds for a unit increase in $\left (x_{ijk} - \bar {x}_{jk} \right)$. We place a non-informative Normal distribution on the within-trial covariate effect parameter *β*^*W*^∼*N*(0,10^2^). We label this method *ABadjEb*.

### Implementation and software

All methods were applied by fitting models in Stan [54], using Markov chain Monte Carlo (MCMC) sampling to estimate Bayesian posterior distributions for model parameters. Each implementation consisted of four MCMC chains, where 1,000 iterations were used as an initial burn-in period for each chain. Posterior estimates were based on 1,000 samples per chain, and checked for sensitivity to changes in initial values. The effective sample size and $\hat {R}$ statistics were extracted for an initial assessment to identify signs of chain non-convergence, and further checks were made by inspecting trace and autocorrelation plots [55].

## Results

In this section, we report the results from applying the methods described in “Methods” section to the datasets described in “Applied example: assessing biologics for treating rheumatoid arthritis (RA)” section. All methods were applied to both the original dataset (consisting of 14 RCTs) and the artificial dataset (consisting of two SATs and 12 RCTs from the original dataset). In tables and figures, where a method was applied to the original dataset it is labelled with the suffix *1*, and where it was applied to the artificial dataset it is labelled with the suffix *2* (e.g. the contrast-based unadjusted method with independent baseline response parameters applied to the original dataset is labelled *CBunadjInd1*). Under the contrast-based parametrisation, the methods require a reference treatment to be specified for which we chose placebo. We report the results for the methods without covariates first, and then report the results from applying the methods with adjustment on the baseline response using RA duration (years) as a covariate.

### Methods without covariates

#### Contrast-based methods

**Fig. 2:**
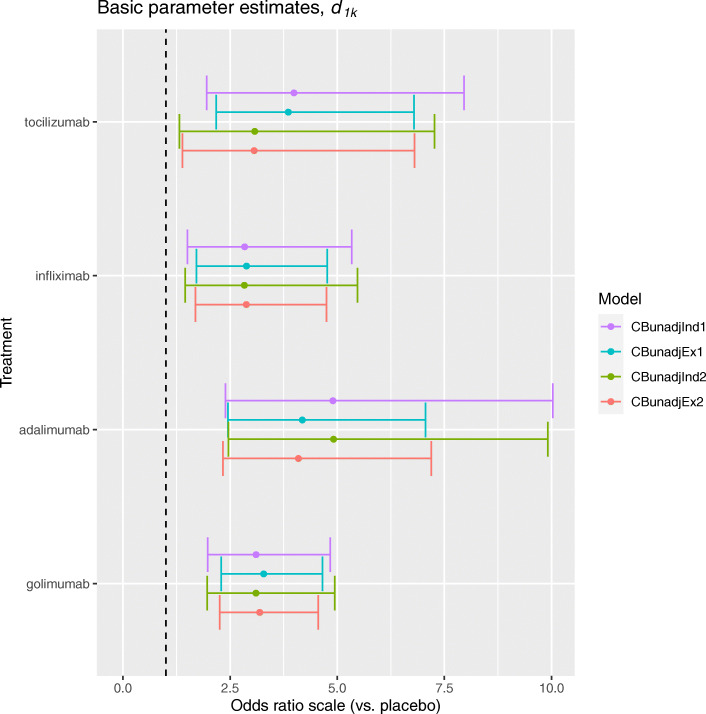
Posterior median and 95% credible interval estimates for log odds ratios comparing each biologic versus placebo in terms of the ACR20 outcome, for contrast-based methods without covariates

The contrast-based methods without adjustment for covariates are described in “Methods without covariates” section, and consist of the methods with independent and exchangeable baseline response parameters, *CBunadjInd* and *CBunadjEx*, respectively. Figure 2 presents the posterior median and 95% credible interval (CrI) estimates for the basic parameters *d*_1*k*_, representing log odds ratios (ORs) comparing biologics versus placebo in terms of the ACR20 outcome, for each method when applied to the original and artificial datasets.

Considering the application to the original dataset first, the *CBunadjInd* method estimates the OR comparing tocilizumab versus placebo to be 3.94 (95% CrI: 1.99, 8.25). This indicates that, in the population represented by the set of trials in the synthesis, a random sample of participants allocated tocilizumab have on average almost four times the odds of achieving an ACR20 response compared to a similar sample of participants allocated placebo. When the *CBunadjInd* method was applied to the artificial dataset, using a two-stage approach (see final paragraph in “Methods without covariates” section), the OR was estimated to be 3.10 (1.34, 6.82). This suggests that the effectiveness of tocilizumab is underestimated in the artificial dataset compared to the original dataset, albeit with significant overlap in CrIs. This result can be explained by the difference in the observed baseline response in the original dataset and the baseline response predicted by the *CBunadjInd* method when applied to the artificial dataset. Figure A.1 (Appendix A) presents the posterior estimates of the baseline response parameters *ϕ*_*j*_ representing the log odds of achieving an ACR20 response on placebo in each trial. For trials one and two, assessing tocilizumab versus placebo, the predicted baseline response overestimates the observed baseline response in the original dataset, which inversely impacts the basic parameter estimates.

Considering the application to the original dataset, the *CBunadjEx* method estimates the OR comparing tocilizumab versus placebo to be 3.86 (2.20, 6.89). This estimate is in agreement with the estimate from the *CBunadjInd* method applied to the original dataset, indicating that the exchangeability assumption had little impact on the baseline response estimates. Figure A.1 (Appendix A) confirms that there is little difference in baseline response estimates between *CBunadjInd* and *CBunadjEx* for trials one and two. When applied to the artificial dataset, *CBunadjEx* estimates the OR to be 3.10 (1.40, 6.96). This result is consistent with the estimate from applying *CBunadjInd* to the artificial dataset using the two-stage approach, but underestimates the OR in the original dataset. This suggests that the disagreement in the effect estimates for tocilizumab is due to the difference between the observed and predicted baseline response estimates, and that the exchangeability assumption had little impact on the results. There is significant overlap in the CrIs corresponding to the effect estimates across all applications, which indicates that the disagreement introduced by using data from SATs (i.e. removing data from an RCT control arm) is relatively limited for this example. Figure 2 also shows that uncertainty increases in treatment effect estimates corresponding to tocilizumab when the control arms are removed, but there is no change in uncertainty for the effect estimates corresponding to the other treatments.

Figure 2 suggests that the exchangeability assumption has a greater impact on effect estimates for adalimumab. Considering the application to the original dataset, the *CBunadjInd* method estimates an OR of 4.90 (2.39, 9.78) for adalimumab versus placebo. In contrast, the *CBunadjEx* method estimates an OR of 4.18 (2.44, 7.17). A similar disagreement is observed in the artificial dataset where the *CBunadjInd* method estimates an OR of 4.81 (2.34, 9.78), whilst *CBunadjEx* estimates an OR of 4.10 (2.34, 6.96). This indicates that the exchangeability assumption introduces a disagreement in the effect estimates for adalimumab in both datasets. Figure A.1 (Appendix A) shows that the baseline response estimates corresponding to trials assessing adalimumab versus placebo (in particular, trials four and six) are significantly shifted toward the mean baseline response (represented by the dashed-line). This suggests that the exchangeability assumption can lead to disagreement in effect estimates, particularly where there is significant between-study heterogeneity in baseline response estimates. It follows that adjusting the baseline response by including covariates, to account for between-study heterogeneity, could mitigate disagreement in effect estimates introduced by the exchangeability assumption. Despite evidence of disagreement in effect estimates there is significant overlap in CrIs, suggesting that the impact of assuming exchangeability is relatively limited for this example.

#### Arm-based methods

**Fig. 3:**
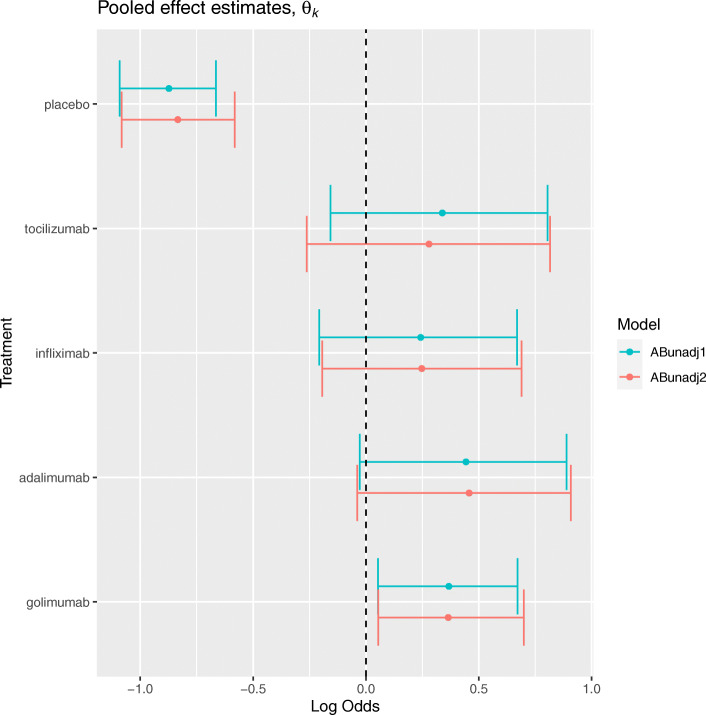
Posterior median and 95% credible interval estimates for the pooled log odds on each intervention in terms of the ACR20 outcome, for arm-based methods without covariates

The arm-based method without covariate-adjustment *ABunadj* is described in “Methods without covariates” section. Figure 3 presents the posterior median and 95% CrI estimates for the pooled absolute effects *θ*_*k*_ representing the log odds of achieving an ACR20 outcome associated with each intervention.

Considering the application to the original dataset first, *ABunadj* estimates the pooled log odds on placebo to be -0.88 (-1.10, -0.67). This implies that, in a population represented by the trials in the synthesis, a random sample of participants allocated placebo would on average have a probability of achieving an ACR20 response of 0.29 (0.25, 0.34). For tocilizumab, the pooled log odds is estimated to be 0.33 (-0.17, 0.81), which corresponds to a response probability of 0.58 (0.46, 0.69). This suggests that tocilizumab is significantly more effective than placebo, in terms of the ACR20 outcome, and is consistent with the results from the contrast-based methods. When *ABunadj* was applied to the artificial dataset, the pooled log odds on placebo was estimated to be -0.83 (-1.09, -0.57), representing a response probability of 0.30 (0.25, 0.36). This is very similar to the estimate of the pooled response probability for *ABunadj* applied to the original dataset. The pooled log odds on tocilizumab is estimated to be 0.28 (-0.26, 0.82), corresponding to a response probability of 0.57 (0.44, 0.69). Here, the result slightly underestimates the effect estimate from the original dataset. This indicates that the removal of data on placebo response, in trials assessing tocilizumab versus placebo, impacted the pooled response estimate for tocilizumab. This may be because the *ABunadj* method assumes that response estimates are correlated at the between-study level, where the correlation *ρ* was estimated to be 0.29 (-0.42, 0.80) for the application to the original dataset, and 0.31 (-0.40, 0.84) for the application to the artificial dataset. There is significant overlap in CrIs for each intervention associated with each dataset, which suggests the impact of the removal of data was limited in this example.

Table B.2 (Appendix B) lists posterior median and 95% CrI estimates for the model parameters corresponding to each application of *ABunadj*. The between-study heterogeneity *τ* was estimated to be 0.34 (0.22, 0.53) when *ABunadj* was applied to the original dataset, which is slightly smaller than the estimate of 0.35 (0.23, 0.55) from the application to the artificial dataset.

### Methods with covariate-adjustment

#### Contrast-based methods

**Fig. 4:**
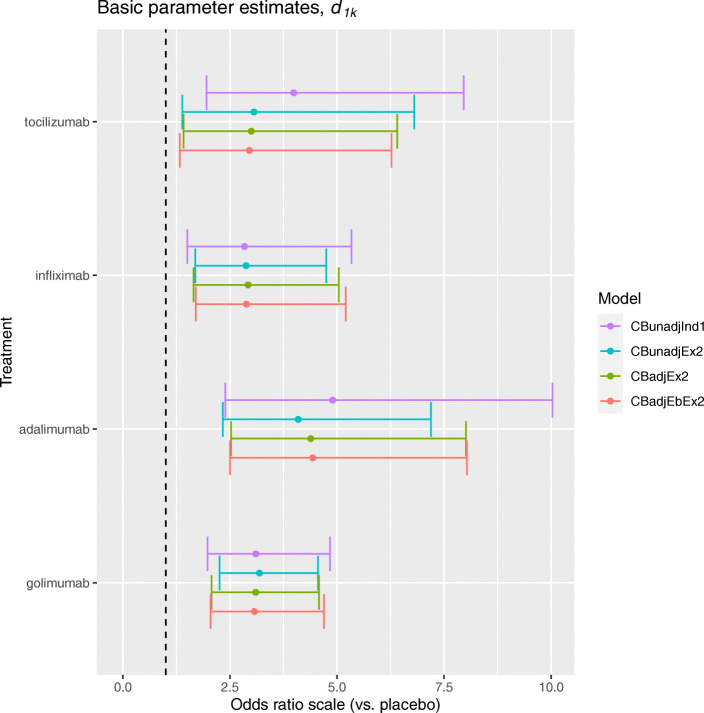
Posterior median and 95% credible interval estimates for log odds ratios comparing each biologic versus placebo in terms of the ACR20 outcome, for contrast ‘-based methods applied to the artificial dataset

In this section, we report the results from applying methods which include an adjustment of the baseline response for RA duration. We begin by considering the results from the contrast-based methods with exchangeable baseline response parameters. Figure 4 presents the posterior median and 95% CrI estimates for the unadjusted and adjusted log ORs, corresponding to the contrast-based methods applied to the artificial dataset.

For tocilizumab, the OR estimated by the *CBadjEx* method, which included adjustment for the mean RA duration, was 3.00 (1.42, 6.42) which suggests that in a trial of newly-diagnosed RA patients (i.e. mean RA duration equal to zero years), participants allocated tocilizumab have approximately three times greater odds of achieving an ACR20 response compared to a similar group of participants allocated placebo. This result underestimates the unadjusted OR 4.10 (2.34, 6.96) estimated by the *CBunadj* method, which represents the average effect of tocilizumab across varying levels of RA duration. This difference in results indicates that the effect of tocilizumab may vary with RA duration, and Table B.3 (Appendix B) confirms the presence of an across trial covariate effect (*α*^*A*^) of -0.10 (-0.20, 0.00). This implies that the effect of a biologic versus placebo in a trial in which a sample of patients have had RA for an average of two years will be approximately 10% (100×(1−*exp*(−0.10))) smaller than in a trial in which a similar sample of patients have had RA for an average of one year.

The *CBadjEbEx* method, which also includes an adjustment for the within-trial covariate effect of RA duration, estimates an OR of 2.94 (1.34, 6.30) comparing tocilizumab versus placebo. This result is in strong agreement with the *CBadjEx* method, which suggests a lack of a covariate effect at the participant-level. Table B.3 (Appendix B) lists the within-trial covariate effect (*α*^*W*^) estimate as 0.00 (-0.02, 0.01). The difference between across- and within-trial covariate effect estimates indicates aggregation bias, and may be due to a difference in the distribution of RA duration within trials and the distribution of mean RA duration across trials. Figure 4 also presents the posterior estimates for the unadjusted log OR (comparing tocilizumab versus placebo) from the contrast-based method with independent baseline response parameters (*CBunadjInd*) applied to the original dataset. The unadjusted OR, estimated in the artificial dataset, underestimates the unadjusted OR estimated in the original dataset, and this difference does not decrease after adjustment for the covariate effect of RA duration. This shows that the adjustment has not mitigated the disagreement in results between the original and artificial datasets. Figure A.2 (Appendix A) presents the posterior estimates for the baseline response corresponding to each method. For trials one and two, there is little difference in the estimates before and after adjustment. This could be due to the fact that RA duration does not explain heterogeneity in the baseline response.

For adalimumab versus placebo, the adjusted OR estimated by the *CBadjEx* method was 4.39 (2.53, 8.00). This estimate is more consistent with the unadjusted OR from the original dataset, estimated by *CBunadjInd* to be 4.90 (2.39, 10.07). In Figure A.2 (Appendix A), the adjusted baseline response estimates in trials four, five, and six (i.e. trials assessing adalimumab versus placebo) show some discrepancy with respect to the unadjusted baseline response estimates from the original dataset.

Table B.3 (Appendix B) shows that there is a reduction in between-study heterogeneity in baseline response (*σ*_*ϕ*_) after adjusting for the mean RA duration, from 0.33 (0.15, 0.60) estimated by *CBunadjEx* to 0.28 (0.12, 0.56) estimated by *CBadjEx*. This indicates that the inclusion of mean RA duration is explaining some of the heterogeneity in baseline response, although there is little change in the mean baseline response *m*_*ϕ*_.

#### Arm-based methods

**Fig. 5:**
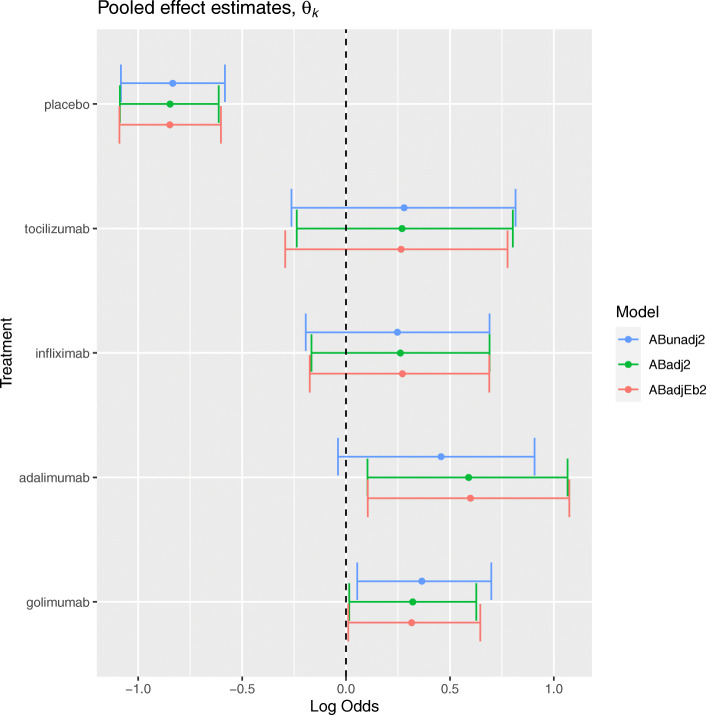
Posterior median and 95% credible interval estimates for the pooled log odds on each intervention in terms of the ACR20 outcome, for arm-based methods applied to the artificial dataset

Figure 5 presents the posterior median and 95% CrI estimates for the unadjusted and adjusted arm-based methods, applied to the artificial dataset. For placebo, the unadjusted pooled log odds of achieving an ACR20 response was estimated to be -0.83 (-1.08, -0.58), corresponding to a response probability of 0.30 (0.25, 0.36). This result was relatively unchanged after adjustment for mean RA duration at the arm-level, where the adjusted pooled log odds were estimated to be -0.85 (-1.09, -0.61) and the corresponding response probability was 0.30 (0.25, 0.36). Similarly, the adjustment did not seem to have an impact on effect estimates for tocilizumab, where the unadjusted pooled log odds were estimated to be 0.28 (-0.26, 0.82) and the adjusted pooled log odds were 0.27 (-0.24, 0.80). In contrast, the effect on adalimumab was larger after adjustment indicating the presence of a covariate effect across trials. The unadjusted pooled log odds of response were 0.59 (0.10, 1.07) corresponding to a response probability of 0.64 (0.52, 0.74), whilst the adjusted pooled log odds of response were 0.46 (-0.04, 0.91) corresponding to a response probability of 0.61 (0.49, 0.71). Table B.4 (Appendix B) lists the posterior estimates for the across-trial covariate effect *β*^*A*^, estimated by the *ABadj* method to be -0.06 (-0.14, 0.02). This suggests that there is a decrease of approximately 6% in the pooled log odds of achieving an ACR20 response, for each additional year a sample of patients spend diagnosed with RA. There does not appear to be evidence of a within-trial covariate effect *β*^*W*^, which is estimated by the *ABadjEb* method to be 0.00 (-0.02, 0.01). Although there does not seem to be a noticeable difference in the between-study heterogeneity (*τ*) before and after adjustment, the correlation *ρ* decreases from 0.31 (-0.40, 0.87) to 0.23 (-0.49, 0.82).

Table B.5 (Appendix B) lists deviance information criterion (DIC) statistics, a measure of model fit [56] where smaller values are preferred, for each method applied to the artificial dataset. There does not seem to be a significant difference in model fit between the arm- and contrast-based methods. The inclusion of RA duration as a covariate did not provide a significant reduction in DIC.

## Discussion

In this paper, we develop contrast- and arm-based methods for NMA synthesising SATs and RCTs, using a mixture of IPD and AD, for a dichotomous outcome. We also extended these methods to adjust the baseline response for a covariate, to account for within- and across-trial covariate effects. We applied the methods to a dataset of 14 RCTs assessing biologics versus placebo for RA and an artificial dataset, including two SATs (assessing tocilizumab), generated from the original dataset of 14 RCTs by removing the control arm in the two tocilizumab trials. Placebo was designated as the reference treatment for the network, and so the contrast-based methods assumed a fixed baseline treatment across trials. The contrast-based unadjusted methods underestimated the treatment effect for tocilizumab versus placebo, when applied to the artificial dataset compared to the original dataset. This difference appeared to be caused by an overestimate of the predicted baseline response for the SATs. The contrast-based methods with exchangeable baseline response parameters showed disagreement with the methods assuming independent parameters, in treatment effect estimates for adalimumab, in both datasets. This disagreement seemed to be caused by a shift toward the mean baseline response associated with the exchangeability assumption for the baseline response parameters. Despite these discrepancies, there was large overlap in posterior distributions for treatment effect parameters between applications to the two datasets. Under both the contrast- and arm-based parametrisation, there was some evidence of an across-trial covariate effect associated with mean RA duration, but a within-trial covariate effect was not detected. However, the adjustment appeared to make little difference to the treatment effect estimates. The arm-based methods showed greater agreement in effect estimates between the applications to the two datasets.

Our applied example enabled an assessment of the developed methods to synthesise SATs and RCTs against a synthesis of RCTs alone. A similar approach was used by Beliveau et al. [27], where connected networks of RCTs were disconnected by removing data from trials, to understand the impact of assuming exchangeable baseline response parameters to connect the disconnected networks. In the majority of cases, the exchangeability assumption seemed to make relatively little difference to the results, as posterior distributions for treatment effect parameters showed large overlap. This is consistent with the findings from our applied example, where there was also significant overlap in posterior distributions for treatment effect parameters in the artificial and original datasets, although the effect estimate for tocilizumab had greater precision in the original dataset where more data were available. Despite this, there was a shift in effect estimates associated with assuming exchangeable baseline response parameters, due to the influence of the mean baseline response (e.g. for adalimumab). To better understand this discrepancy, we also applied the contrast-based method with independent baseline response parameters to the artificial dataset using a two-stage approach. This approach is recommended by Dias et al. [52] to ensure treatment effect estimates are not influenced by assumptions regarding the baseline response. This mitigated the discrepancy associated with assuming exchangeable baseline response parameters, and the results were more consistent with the application to the original dataset for adalimumab. However, the discrepancy remained for treatment effect estimates associated with tocilizumab, indicating that the predicted baseline response in the artificial dataset was not consistent with the observed baseline response in the original dataset. This implies that incorporating SATs into a NMA to estimate relative treatment effects depends significantly on how representative the mean baseline response across the RCTs in the synthesis is of the hypothetical baseline response for the participants in the SATs. This highlights the importance of considering whether each trial in the synthesis is informative about the target population in the SATs [52]. It also suggests that explaining between-study heterogeneity in the baseline response, by including covariates to account for differences in prognostic factors in trial participants, could improve the predicted baseline response. Thus, incorporating SATs into a synthesis may be beneficial when decision-makers require relative effect estimates for a particular treatment, and are reluctant to delay the decision until a RCT has been undertaken. A review of HTA submissions found an increasing use of data from SATs in recent years, particularly to provide evidence on an external patient cohort to determine relative treatment effectiveness [57]. Malottki et al. conducted a multiple technology appraisal of five biologics as second-line treatment for RA [58], in which they identified five RCTs and 28 SATs relevant to the decision problem. Only two biologics were compared head-to-head via an indirect comparison based on two RCTs and the SATs were not included in any quantitative synthesis, resulting in large uncertainty in effectiveness estimates.

When seeking to combine data from SATs and RCTs in a meta-analysis (or NMA), particularly in a decision-making context, it can be beneficial to implement a range of methods to understand how alternative parametrisations influence the results. A comparison of arm- and contrast-based methods requires the calculation of additional quantities as a function of model parameters. For arm-based methods, a pooled relative treatment effect can be calculated from the pooled arm-level outcomes, *d*_1*k*_=*θ*_*k*_−*θ*_1_. For contrast-based methods, a pooled absolute treatment effect can be calculated from the mean baseline response and the pooled relative treatment effect, *θ*_*k*_=*m*_*ϕ*_+*d*_*ik*_.

We also applied methods which included adjustment for a covariate, to account for between-study heterogeneity in baseline response, considering RA duration to be a prognostic factor associated with ACR20 response. In the artificial dataset, an across-trial covariate effect was associated with mean RA duration. The results indicate that the adjustment provided a small reduction in between-study heterogeneity, but this did not make treatment effect estimates more consistent with those from the application to the original dataset. The residual between-study heterogeneity estimated after adjustment suggested the presence of unmeasured prognostic factors. Despite some evidence of an across-trial covariate effect, there was no evidence of a within-trial covariate effect, indicating the presence of aggregation bias. The difference between these findings may be due to the impact of unmeasured covariates, associated with both an ACR20 response and mean RA duration across trials. An exploratory analysis of the IPD, corresponding to the two tocilizumab trials for which it was available (see Table 1), was undertaken to assess the distribution of RA duration across patients within each trial. The distribution of RA duration within each trial did not differ significantly from the distribution of mean RA duration across the other 12 RCTs (listed in Table 1). Here, the goal of the adjustment for RA duration was to use data from RCTs to predict baseline response estimates more representative (compared to the unadjusted estimates) of the patient population in the SATs. Whilst we focus on adjustment on the baseline response, other authors have looked at how a treatment effect varies with respect to a trial characteristic (treatment-covariate interaction) [59], via meta-regression or network meta-regression. Population-adjustment methods, such as matching-adjustment indirect comparison and simulated treatment comparison methods, have been proposed for a similar goal where IPD are available for one trial and only AD are available for another [16]. Similarly, NMA methods have been developed which combine IPD and AD whilst accounting for a non-linear association between the treatment effect and a particular covariate to mitigate aggregation bias [60]. More recently, Hong et al. propose NMA methods using AD to define a prior distribution for pooled treatment effect and treatment-covariate interaction parameters estimated from IPD, whilst down-weighting the AD via a power term or a commensurability parameter, which also avoid introducing aggregation bias into the synthesis [61].

Due to the limited data available in the artificial dataset, we assumed a common within-trial covariate effect parameter. An assumption of independent within-trial covariate effects may have accounted for the difference in covariate distributions between the two trials for which IPD were available. Thom et al. were unable to detect across-trial covariate effects in a similar application due to limited data [20]. In our applied example, there were no trials comparing biologics head-to-head, preventing an assessment of inconsistency via the recommended methods [62]. Due to this, and the limited data, we did not consider adjustment of treatment effects for potential effect modifiers.

In this paper, we made the assumption that the intervention arms in RCTs assessing tocilizumab versus placebo were representative of SATs assessing tocilizumab. In practice, the populations represented by the trials may differ due to factors associated with trial design (e.g. participant inclusion criteria). A better assessment of the methods would be provided by a simulation study, where data are simulated for a network of RCTs and then a subset of data corresponding to participants in control arms are removed, to provide a more accurate representation of data from SATs. This would allow the methods to be applied in a greater number of scenarios to understand how differences in trial populations can influence treatment effect estimates when incorporating SATs into a network. Furthermore, many of the trials considered in the applied example consisted of multiple intervention arms, from which data were combined to create a single intervention arm. The proposed methods can be extended for application to multi-arm RCTs, by considering within-trial correlation between treatment effects relative to a common baseline treatment [51].

## Conclusion

In this paper, we propose contrast- and arm-based NMA methods which allow the incorporation of SATs into a synthesis of RCTs, to estimate relative treatment effectiveness when there is no alternative (due to a disconnected network). Further work is required to understand how adjustment for covariate effects can be incorporated to predict a more representative baseline response estimate for SATs, and treatment effect estimates more consistent with randomised evidence.

## Supplementary Information

**Additional file 1** Supplementary material for: bayesian network meta-analysis methods for combining individual participant data and aggregate data from single arm trials and randomised controlled trials. Includes: Figures A.1-2, Tables B.1-5, Stan programming code for models.

## Data Availability

The datasets analysed during the study are included in this published article (Table 1, applicable only to data extracted from published manuscripts). For eligible studies qualified researchers may request access to individual patient level clinical data through a data request platform. At the time of writing this request platform is Vivli (https://vivli.org/ourmember/roche/). For up to date details on Roche’s Global Policy on the Sharing of Clinical Information and how to request access to related clinical study documents, see here: https://go.roche.com/data_sharing.

## Abbreviations

NMA: Network meta-analysis
SAT: Single arm trial
IPD: Individual participant data
AD: Aggregate data
RCT: Randomised controlled trial
CB: Contrast based
AB: Arm based
RA: Rheumatoid arthritis
HTA: Health technology assessment
bDMARD: biologic disease modifying anti rheumatic drug
ACR: American College of Rheumatology
MCMC: Markov chain Monte Carlo
CrI: Credible interval
OR: Odds ratio
DIC: Deviance information criterion
