# Supplementary material for: Bayesian network meta-analysis methods for combining individual participant data and aggregate data from single arm trials and randomised controlled trials
Source: BMC Med Res Methodol. 2022 Jul 11;22:186. doi: 10.1186/s12874-022-01657-y (PMC9275254; doi:10.1186/s12874-022-01657-y)
Supplement: Supplementary file 1 — Additional file 1 Supplementary material for: bayesian network meta-analysis methods for combining individual participant data and aggregate data from single arm trials and randomised controlled trials. Includes: Figures A.1-2, Tables B.1-5, Stan programming code for models. [file 12874_2022_1657_MOESM1_ESM.pdf]

Supplementary material for: *Bayesian network meta-analysis methods for combining individual participant data and aggregate data from single-arm trials and randomised controlled trials*

Janharpreet Singh*[1], Sandro Gsteiger[2], Lorna Wheaton[1], Richard D. Riley[3], Keith R. Abrams[4], Clare L. Gillies[5], and Sylwia Bujkiewicz[1]

[1]Biostatistics Research Group, Department of Health Sciences, University of Leicester, Leicester, UK
[2]F. Hoffmann-La Roche Ltd, Basel, Switzerland
[3]Centre for Prognosis Research, School of Medicine, Keele University, Staffordshire, UK
[4]Department of Statistics, University of Warwick, Coventry, UK
[5]Leicester Real World Evidence Unit, Leicester Diabetes Centre, Leicester, UK

*Corresponding author: js929@leicester.ac.uk

Appendix A - Additional figures

Baseline response estimates for contrast-based unadjusted methods

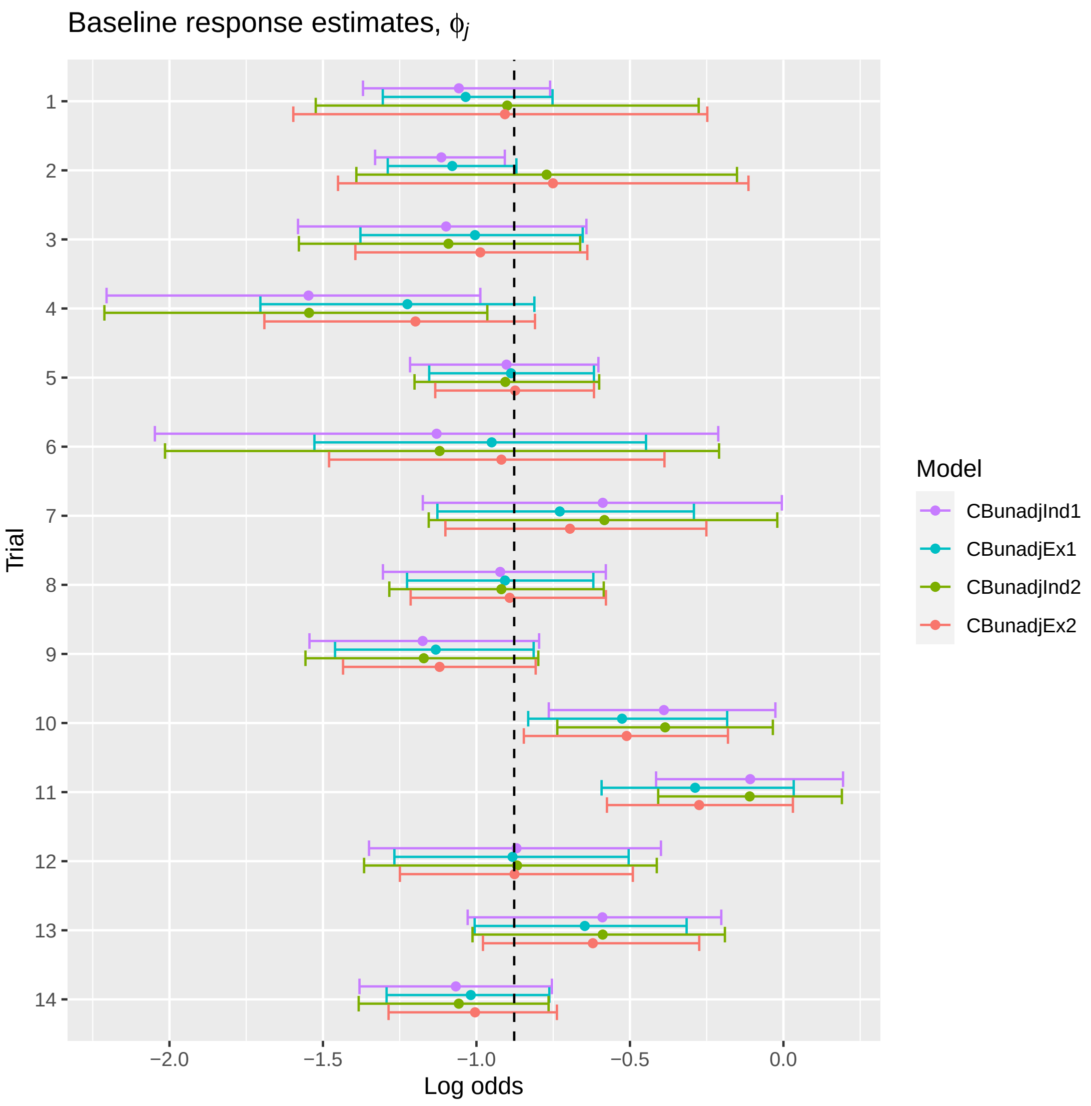

Figure A1: Dashed-line represents mean baseline response m_ϕ from applying *CBunadjEx1*.

Baseline response estimates for contrast-based methods applied to the artificial dataset

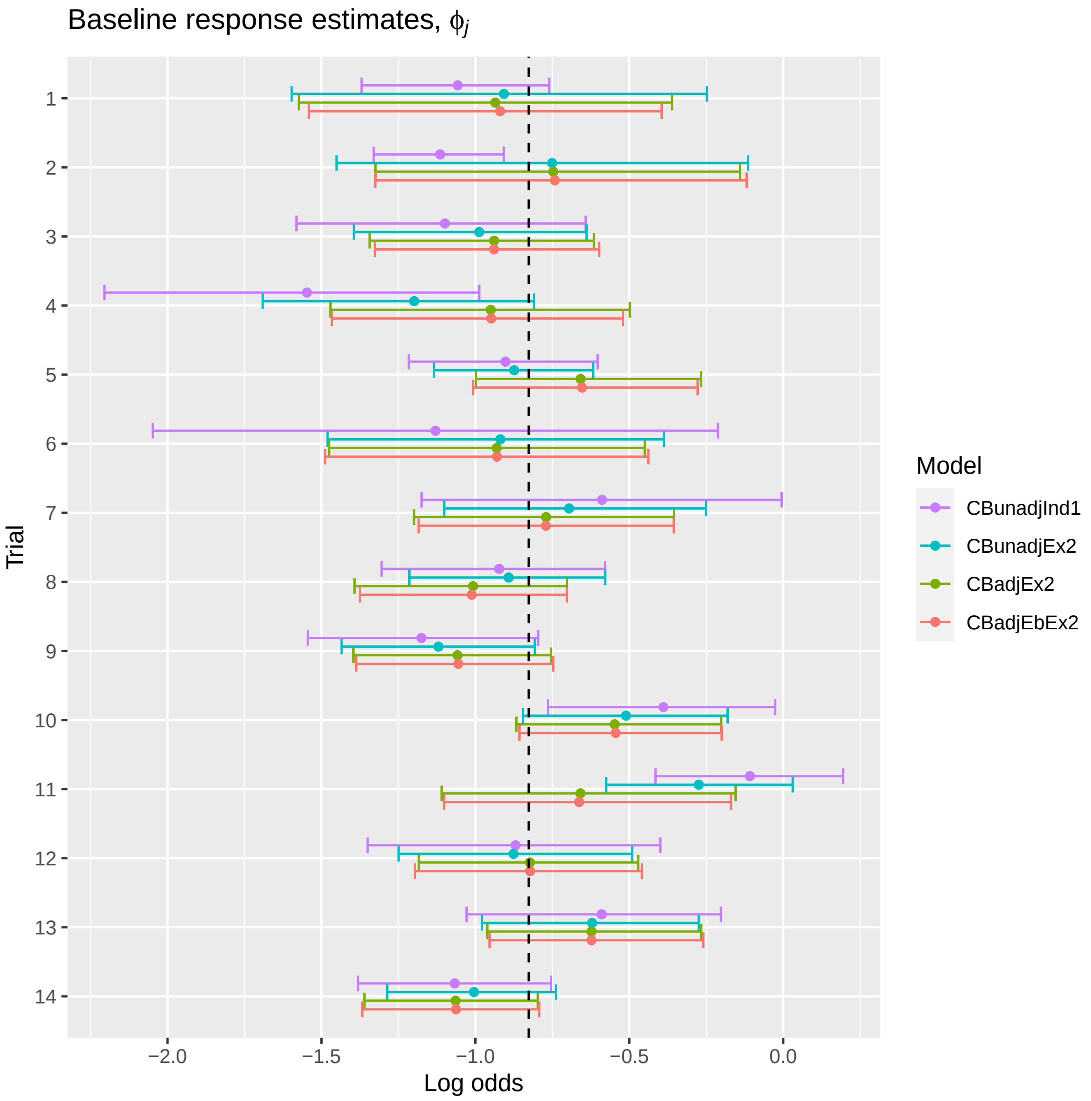

Figure A2: Dashed-line represents mean baseline response m_ϕ from applying *CBunadjEx2*.

Appendix B - Additional tables

Parameter estimates for contrast-based unadjusted methods

Table B1: Median and 95% credible interval estimates for model parameters corresponding to the contrast-based unadjusted methods.

Parameter	CBunadjInd1	CBunadjEx1	CBunadjInd2	CBunadjEx2
ϕ_1	-1.06 (-1.37, -0.76)	-1.04 (-1.31, -0.75)	-0.90 (-1.52, -0.28)	-0.91 (-1.60, -0.25)
ϕ_2	-1.11 (-1.33, -0.91)	-1.08 (-1.29, -0.87)	-0.77 (-1.39, -0.15)	-0.75 (-1.45, -0.11)
ϕ_3	-1.10 (-1.58, -0.64)	-1.00 (-1.38, -0.65)	-1.09 (-1.58, -0.66)	-0.99 (-1.39, -0.64)
ϕ_4	-1.55 (-2.20, -0.99)	-1.22 (-1.70, -0.81)	-1.55 (-2.21, -0.96)	-1.20 (-1.69, -0.81)
ϕ_5	-0.90 (-1.22, -0.60)	-0.89 (-1.15, -0.62)	-0.91 (-1.20, -0.60)	-0.87 (-1.13, -0.62)
ϕ_6	-1.13 (-2.05, -0.21)	-0.95 (-1.53, -0.45)	-1.12 (-2.01, -0.21)	-0.92 (-1.48, -0.39)
ϕ_7	-0.59 (-1.17, -0.01)	-0.73 (-1.13, -0.29)	-0.58 (-1.16, -0.02)	-0.70 (-1.10, -0.25)
ϕ_8	-0.92 (-1.30, -0.58)	-0.91 (-1.23, -0.62)	-0.92 (-1.28, -0.59)	-0.89 (-1.21, -0.58)
ϕ_9	-1.18 (-1.54, -0.80)	-1.13 (-1.46, -0.81)	-1.17 (-1.56, -0.80)	-1.12 (-1.43, -0.81)
ϕ_{10}	-0.39 (-0.76, -0.03)	-0.53 (-0.83, -0.18)	-0.39 (-0.74, -0.03)	-0.51 (-0.85, -0.18)
ϕ_{11}	-0.11 (-0.41, 0.19)	-0.29 (-0.59, 0.03)	-0.11 (-0.41, 0.19)	-0.27 (-0.57, 0.03)
ϕ_{12}	-0.87 (-1.35, -0.40)	-0.88 (-1.27, -0.50)	-0.87 (-1.37, -0.41)	-0.88 (-1.25, -0.49)
ϕ_{13}	-0.59 (-1.03, -0.20)	-0.65 (-1.01, -0.32)	-0.59 (-1.01, -0.19)	-0.62 (-0.98, -0.27)
ϕ_{14}	-1.07 (-1.38, -0.75)	-1.02 (-1.29, -0.76)	-1.06 (-1.38, -0.77)	-1.00 (-1.29, -0.74)
d_1	0.00 (0.00, 0.00)	0.00 (0.00, 0.00)	0.00 (0.00, 0.00)	0.00 (0.00, 0.00)
d_2	1.38 (0.67, 2.07)	1.35 (0.78, 1.92)	1.12 (0.27, 1.98)	1.12 (0.33, 1.92)
d_3	1.04 (0.41, 1.67)	1.06 (0.54, 1.56)	1.04 (0.37, 1.70)	1.06 (0.52, 1.56)
d_4	1.59 (0.87, 2.31)	1.43 (0.90, 1.95)	1.59 (0.90, 2.29)	1.41 (0.85, 1.97)
d_5	1.13 (0.68, 1.58)	1.19 (0.83, 1.54)	1.13 (0.68, 1.60)	1.16 (0.81, 1.52)
σ	0.42 (0.20, 0.81)	0.32 (0.14, 0.64)	0.42 (0.19, 0.84)	0.32 (0.10, 0.70)

Parameter estimates for arm-based unadjusted methods

Table B2: Median and 95% credible interval estimates for model parameters corresponding to the arm-based unadjusted models.

Parameter	ABunadj1	ABunadj2
θ_1	-0.87 (-1.09, -0.66)	-0.83 (-1.08, -0.58)
θ_2	0.34 (-0.16, 0.80)	0.28 (-0.26, 0.82)
θ_3	0.24 (-0.21, 0.67)	0.25 (-0.19, 0.69)
θ_4	0.44 (-0.03, 0.89)	0.46 (-0.04, 0.91)
θ_5	0.37 (0.05, 0.67)	0.36 (0.05, 0.70)
ν_1	-0.13 (-0.48, 0.19)	-0.14 (-0.68, 0.39)
ν_2	-0.20 (-0.66, 0.29)	0.15 (-0.39, 0.71)
ν_3	0.09 (-0.37, 0.60)	0.04 (-0.42, 0.49)
ν_4	-0.22 (-0.52, 0.06)	-0.23 (-0.68, 0.20)
ν_5	0.04 (-0.39, 0.51)	-0.05 (-0.53, 0.44)
ν_6	-0.20 (-0.64, 0.19)	-0.46 (-0.99, -0.01)
ν_7	-0.04 (-0.50, 0.45)	0.03 (-0.42, 0.53)
ν_8	-0.43 (-0.97, 0.03)	-0.04 (-0.38, 0.31)
ν_9	0.04 (-0.41, 0.53)	-0.14 (-0.70, 0.41)
ν_{10}	0.00 (-0.33, 0.33)	0.00 (-0.63, 0.60)
ν_{11}	-0.12 (-0.66, 0.39)	0.08 (-0.32, 0.49)
ν_{12}	0.01 (-0.58, 0.58)	0.15 (-0.34, 0.66)
ν_{13}	0.08 (-0.32, 0.49)	0.02 (-0.38, 0.38)
ν_{14}	0.16 (-0.33, 0.68)	-0.08 (-0.47, 0.29)
ν_{15}	0.02 (-0.35, 0.37)	-0.54 (-0.93, -0.19)
ν_{16}	-0.06 (-0.43, 0.32)	-0.25 (-0.64, 0.15)
ν_{17}	-0.54 (-0.90, -0.19)	0.13 (-0.33, 0.59)
ν_{18}	-0.21 (-0.60, 0.18)	0.36 (0.00, 0.77)
ν_{19}	0.14 (-0.32, 0.60)	0.13 (-0.26, 0.50)
ν_{20}	0.40 (0.03, 0.78)	0.64 (0.30, 1.03)
ν_{21}	0.12 (-0.25, 0.49)	-0.13 (-0.67, 0.34)
ν_{22}	0.67 (0.34, 1.04)	-0.01 (-0.44, 0.42)
ν_{23}	-0.13 (-0.62, 0.34)	0.49 (0.11, 0.90)
ν_{24}	0.01 (-0.41, 0.43)	0.13 (-0.30, 0.56)
ν_{25}	0.48 (0.10, 0.90)	-0.03 (-0.38, 0.32)
ν_{26}	0.15 (-0.26, 0.56)	-0.21 (-0.58, 0.13)
ν_{27}	-0.03 (-0.37, 0.33)	
ν_{28}	-0.18 (-0.51, 0.14)	
τ	0.34 (0.22, 0.53)	0.35 (0.23, 0.55)
ρ	0.29 (-0.42, 0.80)	0.31 (-0.40, 0.82)

Parameter estimates for contrast-based methods applied to the artificial dataset

Table B3: Median and 95% credible interval estimates for model parameters corresponding to the contrast-based unadjusted and adjusted methods.

Parameter	CBunadjEx2	CBadjEx2	CBadjEbEx2
m_ϕ	-0.83 (-1.08, -0.59)	-0.84 (-1.07, -0.62)	-0.83 (-1.06, -0.62)
σ_ϕ	0.33 (0.18, 0.60)	0.28 (0.12, 0.56)	0.27 (0.12, 0.56)
ϕ_1	-0.91 (-1.60, -0.25)	-0.94 (-1.57, -0.36)	-0.92 (-1.54, -0.39)
ϕ_2	-0.75 (-1.45, -0.11)	-0.75 (-1.32, -0.14)	-0.74 (-1.33, -0.12)
ϕ_3	-0.99 (-1.39, -0.64)	-0.94 (-1.34, -0.62)	-0.94 (-1.33, -0.60)
ϕ_4	-1.20 (-1.69, -0.81)	-0.95 (-1.47, -0.50)	-0.95 (-1.47, -0.52)
ϕ_5	-0.87 (-1.13, -0.62)	-0.66 (-1.00, -0.27)	-0.65 (-1.01, -0.28)
ϕ_6	-0.92 (-1.48, -0.39)	-0.93 (-1.47, -0.45)	-0.93 (-1.49, -0.44)
ϕ_7	-0.70 (-1.10, -0.25)	-0.77 (-1.20, -0.35)	-0.77 (-1.18, -0.36)
ϕ_8	-0.89 (-1.21, -0.58)	-1.01 (-1.39, -0.70)	-1.01 (-1.38, -0.70)
ϕ_9	-1.12 (-1.43, -0.81)	-1.06 (-1.40, -0.75)	-1.06 (-1.39, -0.75)
ϕ_{10}	-0.51 (-0.85, -0.18)	-0.55 (-0.87, -0.20)	-0.54 (-0.86, -0.20)
ϕ_{11}	-0.27 (-0.57, 0.03)	-0.66 (-1.11, -0.15)	-0.66 (-1.10, -0.17)
ϕ_{12}	-0.88 (-1.25, -0.49)	-0.82 (-1.18, -0.47)	-0.82 (-1.20, -0.46)
ϕ_{13}	-0.62 (-0.98, -0.27)	-0.62 (-0.96, -0.27)	-0.62 (-0.95, -0.26)
ϕ_{14}	-1.00 (-1.29, -0.74)	-1.06 (-1.36, -0.80)	-1.06 (-1.37, -0.79)
d_1	0.00 (0.00, 0.00)	0.00 (0.00, 0.00)	0.00 (0.00, 0.00)
d_2	1.12 (0.33, 1.92)	1.10 (0.35, 1.86)	1.08 (0.29, 1.84)
d_3	1.06 (0.52, 1.56)	1.07 (0.50, 1.62)	1.06 (0.53, 1.65)
d_4	1.41 (0.85, 1.97)	1.48 (0.93, 2.08)	1.49 (0.92, 2.08)
d_5	1.16 (0.81, 1.52)	1.13 (0.73, 1.52)	1.12 (0.72, 1.55)
σ	0.32 (0.10, 0.70)	0.36 (0.16, 0.73)	0.37 (0.16, 0.75)
α^A		-0.10 (-0.20, 0.00)	-0.10 (-0.21, 0.00)
α^W			0.00 (-0.02, 0.01)

Parameter estimates for arm-based methods applied to the artificial dataset

Table B4: Median and 95% credible interval estimates for model parameters corresponding to the arm-based unadjusted and adjusted models.

Parameter	ABunadj2	ABadj2	ABadjEb2
θ_1	-0.83 (-1.08, -0.58)	-0.85 (-1.09, -0.61)	-0.85 (-1.09, -0.60)
θ_2	0.28 (-0.26, 0.82)	0.27 (-0.24, 0.80)	0.26 (-0.29, 0.78)
θ_3	0.25 (-0.19, 0.69)	0.26 (-0.17, 0.69)	0.27 (-0.17, 0.69)
θ_4	0.46 (-0.04, 0.91)	0.59 (0.10, 1.07)	0.60 (0.10, 1.07)
θ_5	0.36 (0.05, 0.70)	0.32 (0.02, 0.63)	0.32 (0.01, 0.65)
ν_1	-0.14 (-0.68, 0.39)	-0.22 (-0.76, 0.31)	-0.22 (-0.77, 0.33)
ν_2	0.15 (-0.39, 0.71)	0.21 (-0.32, 0.72)	0.22 (-0.30, 0.77)
ν_3	0.04 (-0.42, 0.49)	0.04 (-0.39, 0.48)	0.04 (-0.37, 0.49)
ν_4	-0.23 (-0.68, 0.20)	-0.18 (-0.60, 0.22)	-0.18 (-0.64, 0.24)
ν_5	-0.05 (-0.53, 0.44)	0.09 (-0.41, 0.62)	0.08 (-0.40, 0.58)
ν_6	-0.46 (-0.99, -0.01)	-0.31 (-0.90, 0.15)	-0.32 (-0.91, 0.19)
ν_7	0.03 (-0.42, 0.53)	0.09 (-0.38, 0.57)	0.08 (-0.37, 0.53)
ν_8	-0.04 (-0.38, 0.31)	0.12 (-0.26, 0.51)	0.12 (-0.29, 0.56)
ν_9	-0.14 (-0.70, 0.41)	-0.23 (-0.81, 0.32)	-0.24 (-0.85, 0.27)
ν_{10}	0.00 (-0.63, 0.60)	-0.02 (-0.62, 0.60)	-0.01 (-0.62, 0.60)
ν_{11}	0.08 (-0.32, 0.49)	0.11 (-0.27, 0.53)	0.12 (-0.27, 0.51)
ν_{12}	0.15 (-0.34, 0.66)	0.09 (-0.40, 0.57)	0.09 (-0.39, 0.59)
ν_{13}	0.02 (-0.38, 0.38)	-0.07 (-0.46, 0.31)	-0.07 (-0.44, 0.31)
ν_{14}	-0.08 (-0.47, 0.29)	-0.14 (-0.54, 0.25)	-0.14 (-0.54, 0.22)
ν_{15}	-0.54 (-0.93, -0.19)	-0.45 (-0.85, -0.10)	-0.45 (-0.86, -0.09)
ν_{16}	-0.25 (-0.64, 0.15)	-0.24 (-0.63, 0.14)	-0.24 (-0.63, 0.13)
ν_{17}	0.13 (-0.33, 0.59)	0.09 (-0.38, 0.54)	0.08 (-0.36, 0.54)
ν_{18}	0.36 (0.00, 0.77)	0.39 (0.02, 0.78)	0.38 (0.00, 0.79)
ν_{19}	0.13 (-0.26, 0.50)	-0.09 (-0.52, 0.37)	-0.08 (-0.54, 0.35)
ν_{20}	0.64 (0.30, 1.03)	0.39 (-0.07, 0.89)	0.39 (-0.06, 0.90)
ν_{21}	-0.13 (-0.67, 0.34)	-0.06 (-0.58, 0.40)	-0.08 (-0.56, 0.41)
ν_{22}	-0.01 (-0.44, 0.42)	-0.01 (-0.43, 0.39)	-0.01 (-0.43, 0.40)
ν_{23}	0.49 (0.11, 0.90)	0.54 (0.16, 0.95)	0.54 (0.17, 0.96)
ν_{24}	0.13 (-0.30, 0.56)	0.16 (-0.27, 0.57)	0.16 (-0.27, 0.59)
ν_{25}	-0.03 (-0.38, 0.32)	-0.04 (-0.38, 0.29)	-0.04 (-0.39, 0.29)
ν_{26}	-0.21 (-0.58, 0.13)	-0.25 (-0.59, 0.09)	-0.24 (-0.60, 0.08)
τ	0.35 (0.23, 0.55)	0.33 (0.21, 0.55)	0.34 (0.21, 0.54)
ρ	0.31 (-0.40, 0.82)	0.23 (-0.49, 0.82)	0.24 (-0.50, 0.80)
β^A		-0.06 (-0.14, 0.02)	-0.06 (-0.14, 0.02)
β^W			0.00 (-0.02, 0.01)

Deviance information criterion (DIC) statistics for methods applied to artificial dataset

Table B5: DIC statistics, by likelihood, for the constrast- and arm-based models applied to the artificial dataset, 1220 IPD data points and 24 AD data points.

Model	Likelihood	$\bar{D}$	pD	DIC
CBunadjEx2	bernoulli	1652.26	0.65	1652.91
CBadjEx2	bernoulli	1653.93	2.29	1656.22
CBadjEbEx2	bernoulli	1652.35	1.08	1653.43
ABunadj2	bernoulli	1651.67	0.04	1651.70
ABadj2	bernoulli	1653.69	2.03	1655.72
ABadjEb2	bernoulli	1654.79	3.50	1658.29
CBunadjEx2	binomial	27.53	18.07	45.60
CBadjEx2	binomial	25.55	18.11	43.65
CBadjEbEx2	binomial	25.57	18.16	43.73
ABunadj2	binomial	24.27	18.57	42.85
ABadj2	binomial	24.27	18.69	42.97
ABadjEb2	binomial	24.20	18.69	42.89

Appendix C - Stan program code

Contrast-based unadjusted method with exchangeable baseline response parameters (*CBunadjEx*)

```
data{
  // IPD
  int <lower=1>  n_patients_ipd;
  int <lower=1>  study_ipd[n_patients_ipd];
  int <lower=1>  treatment_ipd[n_patients_ipd];
  int <lower=1>  baseline_ipd[n_patients_ipd];
  int <lower=1>  arm_ipd[n_patients_ipd];
  int <lower=0, upper=1>  index_ipd[n_patients_ipd];
  int <lower=0>  Y[n_patients_ipd];
  // AD
  int <lower=1>  n_arms_ad;
  int <lower=1>  study_ad[n_arms_ad];
  int <lower=1>  treatment_ad[n_arms_ad];
  int <lower=1>  baseline_ad[n_arms_ad];
  int <lower=0, upper=1>  index_ad[n_arms_ad];
  int <lower=1>  n[n_arms_ad];
  int <lower=0>  r[n_arms_ad];
  // Prior parameters
  real <lower=0>  prior_norm_sd;
  real <lower=0>  prior_unif_upper;
}

transformed data{
  int <lower=2> n_treatments = max(append_array(treatment_ipd, treatment_ad));
  // IPD
  int <lower=2> n_studies_ipd = max(study_ipd);
  int <lower=2> n_arms_ipd = max(arm_ipd);
  // AD
  int <lower=2> n_studies_ad = max(study_ad);
}

parameters{
  vector[n_studies_ipd + n_studies_ad] u_phi;
  vector[n_arms_ipd + n_arms_ad] u_delta;
  vector[n_treatments - 1] d_est;
  real <lower=0> sigma;
  real m_phi;
  real <lower=0> sigma_phi;
}


transformed parameters{
  vector[n_studies_ipd + n_studies_ad] phi;
```

```
vector[n_treatments] d;
vector[n_arms_ipd + n_arms_ad] m_delta;
vector[n_arms_ipd + n_arms_ad] delta;
vector[n_patients_ipd] lin_pred_ipd;
vector[n_arms_ad] lin_pred_ad;

//###########################
// RANDOM BASELINE RESPONSE
//###########################

phi = m_phi + sigma_phi * u_phi;

//###################
// BASIC PARAMETERS
//###################

d[1] = 0;
for (t in 2:n_treatments) d[t] = d_est[t - 1];

//######################
// NETWORK CONSISTENCY
//######################

for(patient in 1:n_patients_ipd){
  m_delta[arm_ipd[patient]] = d[treatment_ipd[patient]] - d[baseline_ipd[patient]];
}

for(arm in 1:n_arms_ad){
  m_delta[n_arms_ipd + arm] = d[treatment_ad[arm]] - d[baseline_ad[arm]];
}

//###########################
// RANDOM TREATMENT EFFECTS
//###########################

for(arm in 1:n_arms_ipd){
  delta[arm] = m_delta[arm] + sigma * u_delta[arm];
}

for(arm in 1:n_arms_ad){
  delta[n_arms_ipd + arm] = m_delta[n_arms_ipd + arm] +
    sigma * u_delta[n_arms_ipd + arm];
}

//####################
// LINEAR PREDICTORS
//####################

for (patient in 1:n_patients_ipd){
```

```
    lin_pred_ipd[patient] = phi[study_ipd[patient]] +
      delta[arm_ipd[patient]] * index_ipd[patient];
  }

  for (arm in 1:n_arms_ad){
    lin_pred_ad[arm] = phi[n_studies_ipd + study_ad[arm]] +
      delta[n_arms_ipd + arm] * index_ad[arm];
  }
}

model{
  // Implicit logit tranformation onto linear predictor scale.
  Y ~ bernoulli_logit(lin_pred_ipd);
  r ~ binomial_logit(n, lin_pred_ad);

  // Random variability for baseline response.
  u_phi ~ std_normal();

  // Random variability for relative effects.
  u_delta ~ std_normal();

  // Prior distributions
  d_est ~ normal(0, prior_norm_sd);
  sigma ~ uniform(0.0001, prior_unif_upper);
  m_phi ~ normal(0, prior_norm_sd);
  sigma_phi ~ uniform(0.0001, prior_unif_upper);
}

generated quantities{
  // Absolute treatment effects.
  vector[n_treatments] theta;

  for (t in 1:n_treatments) theta[t] = m_phi + d[t];

}
```

Contrast-based adjusted method with exchangeable baseline response parameters (*CBadjEx*)

```
data{
  // IPD
  int <lower=1>  n_patients_ipd;
  int <lower=1>  study_ipd[n_patients_ipd];
  int <lower=1>  treatment_ipd[n_patients_ipd];
  int <lower=1>  baseline_ipd[n_patients_ipd];
  int <lower=1>  arm_ipd[n_patients_ipd];
  int <lower=0, upper=1>  index_ipd[n_patients_ipd];
  int <lower=0>  Y[n_patients_ipd];
  // AD
  int <lower=1>  n_arms_ad;
  int <lower=1>  study_ad[n_arms_ad];
  int <lower=1>  treatment_ad[n_arms_ad];
  int <lower=1>  baseline_ad[n_arms_ad];
  int <lower=0, upper=1>  index_ad[n_arms_ad];
  int <lower=1>  n[n_arms_ad];
  int <lower=0>  r[n_arms_ad];
  // Prior parameters
  real <lower=0>  prior_norm_sd;
  real <lower=0>  prior_unif_upper;
  // Covariate matrices
  int <lower=1> n_cov;
  vector[n_cov] X_ipd[n_patients_ipd];
  vector[n_cov] X_ad[n_arms_ad];
}

transformed data{
  int <lower=2> n_treatments = max(append_array(treatment_ipd, treatment_ad));
  // IPD
  int <lower=2> n_studies_ipd = max(study_ipd);
  int <lower=2> n_arms_ipd = max(arm_ipd);
  // AD
  int <lower=2> n_studies_ad = max(study_ad);
}

parameters{
  row_vector[n_cov] alpha;
  vector[n_studies_ipd + n_studies_ad] u_phi;
  vector[n_arms_ipd + n_arms_ad] u_delta;
  vector[n_treatments - 1] d_est;
  real <lower=0> sigma;
  real m_phi;
  real <lower=0> sigma_phi;
}
```

```
transformed parameters{
  vector[n_studies_ipd + n_studies_ad] phi;
  vector[n_treatments] d;
  vector[n_arms_ipd + n_arms_ad] m_delta;
  vector[n_arms_ipd + n_arms_ad] delta;
  vector[n_patients_ipd] lin_pred_ipd;
  vector[n_arms_ad] lin_pred_ad;

  //###########################
  // RANDOM BASELINE RESPONSE
  //###########################

  phi = m_phi + sigma_phi * u_phi;

  //##################
  // BASIC PARAMETERS
  //##################

  d[1] = 0;
  for (t in 2:n_treatments) d[t] = d_est[t - 1];

  //#####################
  // NETWORK CONSISTENCY
  //#####################

  for(patient in 1:n_patients_ipd){
    m_delta[arm_ipd[patient]] = d[treatment_ipd[patient]] - d[baseline_ipd[patient]];
  }

  for(arm in 1:n_arms_ad){
    m_delta[n_arms_ipd + arm] = d[treatment_ad[arm]] - d[baseline_ad[arm]];
  }

  //###########################
  // RANDOM TREATMENT EFFECTS
  //###########################

  for(arm in 1:n_arms_ipd){
    delta[arm] = m_delta[arm] + sigma * u_delta[arm];
  }

  for(arm in 1:n_arms_ad){
    delta[n_arms_ipd + arm] = m_delta[n_arms_ipd + arm] +
      sigma * u_delta[n_arms_ipd + arm];
  }

  //###################
  // LINEAR PREDICTORS
```

```
  //####################

  for (patient in 1:n_patients_ipd){
    lin_pred_ipd[patient] = phi[study_ipd[patient]] +
      alpha * X_ipd[patient] +
      delta[arm_ipd[patient]] * index_ipd[patient];
  }

  for (arm in 1:n_arms_ad){
    lin_pred_ad[arm] = phi[n_studies_ipd + study_ad[arm]] +
      alpha * X_ad[arm] +
      delta[n_arms_ipd + arm] * index_ad[arm];
  }
}

model{
  // Implicit logit tranformation onto linear predictor scale.
  Y ~ bernoulli_logit(lin_pred_ipd);
  r ~ binomial_logit(n, lin_pred_ad);

  // Random variability for baseline response.
  u_phi ~ std_normal();

  // Random variability for relative effects.
  u_delta ~ std_normal();

  // Prior distributions
  d_est ~ normal(0, prior_norm_sd);
  sigma ~ uniform(0.0001, prior_unif_upper);
  m_phi ~ normal(0, prior_norm_sd);
  sigma_phi ~ uniform(0.0001, prior_unif_upper);
  alpha ~ normal(0, prior_norm_sd);
}

generated quantities{
  // Absolute treatment effects.
  vector[n_treatments] theta;

  for (t in 1:n_treatments) theta[t] = m_phi + d[t];

}
```

Contrast-based adjusted ecological bias method with exchangeable baseline response parameters (*CBadjEbEx*)

```
data{
  // IPD
  int <lower=1>  n_patients_ipd;
  int <lower=1>  study_ipd[n_patients_ipd];
  int <lower=1>  treatment_ipd[n_patients_ipd];
  int <lower=1>  baseline_ipd[n_patients_ipd];
  int <lower=1>  arm_ipd[n_patients_ipd];
  int <lower=0, upper=1>  index_ipd[n_patients_ipd];
  int <lower=0>  Y[n_patients_ipd];
  // AD
  int <lower=1>  n_arms_ad;
  int <lower=1>  study_ad[n_arms_ad];
  int <lower=1>  treatment_ad[n_arms_ad];
  int <lower=1>  baseline_ad[n_arms_ad];
  int <lower=0, upper=1>  index_ad[n_arms_ad];
  int <lower=1>  n[n_arms_ad];
  int <lower=0>  r[n_arms_ad];
  // Prior parameters
  real <lower=0>  prior_norm_sd;
  real <lower=0>  prior_unif_upper;
  // Covariate matrices
  int <lower=1> n_cov;
  vector[n_cov] X_ipd[n_patients_ipd];
  vector[n_cov] X_ad[n_arms_ad];
  vector[n_cov] Xbar_ipd[n_patients_ipd];
}

transformed data{
  int <lower=2> n_treatments = max(append_array(treatment_ipd, treatment_ad));
  // IPD
  int <lower=2> n_studies_ipd = max(study_ipd);
  int <lower=2> n_arms_ipd = max(arm_ipd);
  // AD
  int <lower=2> n_studies_ad = max(study_ad);
}

parameters{
  vector[n_studies_ipd + n_studies_ad] u_phi;
  vector[n_arms_ipd + n_arms_ad] u_delta;
  vector[n_treatments - 1] d_est;
  real <lower=0> sigma;
  real m_phi;
  real <lower=0> sigma_phi;
  row_vector[n_cov] alpha_w;
  row_vector[n_cov] alpha_a;
```

```
}

transformed parameters{
  vector[n_studies_ipd + n_studies_ad] phi;
  vector[n_treatments] d;
  vector[n_arms_ipd + n_arms_ad] m_delta;
  vector[n_arms_ipd + n_arms_ad] delta;
  vector[n_patients_ipd] lin_pred_ipd;
  vector[n_arms_ad] lin_pred_ad;

  //############################
  // RANDOM BASELINE RESPONSE
  //############################

  phi = m_phi + sigma_phi * u_phi;

  //##################
  // BASIC PARAMETERS
  //##################

  d[1] = 0;
  for (t in 2:n_treatments) d[t] = d_est[t - 1];

  //#####################
  // NETWORK CONSISTENCY
  //#####################

  for(patient in 1:n_patients_ipd){
    m_delta[arm_ipd[patient]] = d[treatment_ipd[patient]] - d[baseline_ipd[patient]];
  }

  for(arm in 1:n_arms_ad){
    m_delta[n_arms_ipd + arm] = d[treatment_ad[arm]] - d[baseline_ad[arm]];
  }

  //############################
  // RANDOM TREATMENT EFFECTS
  //############################

  for(arm in 1:n_arms_ipd){
    delta[arm] = m_delta[arm] + sigma * u_delta[arm];
  }

  for(arm in 1:n_arms_ad){
    delta[n_arms_ipd + arm] = m_delta[n_arms_ipd + arm] +
      sigma * u_delta[n_arms_ipd + arm];
  }
```

```
  //###################
  // LINEAR PREDICTORS
  //###################

  for (patient in 1:n_patients_ipd){
    lin_pred_ipd[patient] = phi[study_ipd[patient]] +
      alpha_a * Xbar_ipd[patient] +
      alpha_w * (X_ipd[patient] - Xbar_ipd[patient]) +
      delta[arm_ipd[patient]] * index_ipd[patient];
  }

  for (arm in 1:n_arms_ad){
    lin_pred_ad[arm] = phi[n_studies_ipd + study_ad[arm]] +
      alpha_a * X_ad[arm] +
      delta[n_arms_ipd + arm] * index_ad[arm];
  }
}

model{
  // Implicit logit tranformation onto linear predictor scale.
  Y ~ bernoulli_logit(lin_pred_ipd);
  r ~ binomial_logit(n, lin_pred_ad);

  // Random variability for baseline response.
  u_phi ~ std_normal();

  // Random variability for relative effects.
  u_delta ~ std_normal();

  // Prior distributions
  d_est ~ normal(0, prior_norm_sd);
  sigma ~ uniform(0.0001, prior_unif_upper);
  m_phi ~ normal(0, prior_norm_sd);
  sigma_phi ~ uniform(0.0001, prior_unif_upper);
  alpha_a ~ normal(0, prior_norm_sd);
  alpha_w ~ normal(0, prior_norm_sd);
}

generated quantities{
  // Absolute treatment effects.
  vector[n_treatments] theta;

  for (t in 1:n_treatments) theta[t] = m_phi + d[t];

}
```

Arm-based unadjusted method *ABunadj*

```
data{
  // IPD
  int <lower=1>  n_patients_ipd;
  int <lower=1>  study_ipd[n_patients_ipd];
  int <lower=1>  treatment_ipd[n_patients_ipd];
  int <lower=1>  baseline_ipd[n_patients_ipd];
  int <lower=1>  arm_ipd[n_patients_ipd];
  int <lower=0, upper=1>  index_ipd[n_patients_ipd];
  int <lower=0>  Y[n_patients_ipd];
  // AD
  int <lower=1>  n_arms_ad;
  int <lower=1>  study_ad[n_arms_ad];
  int <lower=1>  treatment_ad[n_arms_ad];
  int <lower=1>  baseline_ad[n_arms_ad];
  int <lower=0, upper=1>  index_ad[n_arms_ad];
  int <lower=1>  n[n_arms_ad];
  int <lower=0>  r[n_arms_ad];
  // Prior parameters
  real <lower=0>  prior_norm_sd;
  real <lower=0>  prior_unif_upper;
  //
  int <lower=2> n_studies_ipd;
  int nu_indices_ipd[n_studies_ipd, 2];
}

transformed data{
  int <lower=2> n_treatments = max(append_array(treatment_ipd, treatment_ad));
  int <lower=2> n_arms_ipd = max(arm_ipd);
  int <lower=2> n_studies_ad = max(study_ad);
}

parameters{
  vector[n_arms_ipd + n_arms_ad] u_nu;
  vector[n_treatments] theta;
  real <lower=0> tau;
  real <lower=-1, upper=1> rho;
}


transformed parameters{
  matrix[2, 2] L;
  vector[n_arms_ipd + n_arms_ad] nu;
  vector[n_patients_ipd] lin_pred_ipd;
  vector[n_arms_ad] lin_pred_ad;

  //########################
```

```
  // CHOLESKY DECOMPOSITION
  //##########################

  L[1, 1] = 1;     L[1, 2] = 0;
  L[2, 1] = rho;  L[2, 2] = sqrt(1 - rho ^ 2);

  //############################
  // RANDOM TREATMENT EFFECTS
  //############################

  for(stud in 1:n_studies_ipd){
    int s = nu_indices_ipd[stud, 1];
    int t = nu_indices_ipd[stud, 2];
    if (s == t) {                    // single-arm trials
      nu[s] = tau * u_nu[s];
    } else {                         // randomised trials
      nu[s:t] = tau * L * u_nu[s:t];
    }
  }

  for(stud in 1:n_studies_ad){
    int s = n_arms_ipd + (2 * stud - 1);
    nu[s:s + 1] = tau * L * u_nu[s:s + 1];
  }

  //####################
  // LINEAR PREDICTORS
  //####################

  for (patient in 1:n_patients_ipd){
    lin_pred_ipd[patient] = theta[treatment_ipd[patient]] + nu[arm_ipd[patient]];
  }

  for (arm in 1:n_arms_ad){
    lin_pred_ad[arm] = theta[treatment_ad[arm]] + nu[n_arms_ipd + arm];
  }
}

model{
  // Implicit logit tranformation onto linear predictor scale.
  Y ~ bernoulli_logit(lin_pred_ipd);
  r ~ binomial_logit(n, lin_pred_ad);

  // Random variability for random effects.
  u_nu ~ std_normal();

  // Prior distributions
  theta ~ normal(0, prior_norm_sd);
  tau ~ uniform(0.0001, prior_unif_upper);
```

```
  rho ~ uniform(-0.999, 0.999);
}

generated quantities{
  // Basic parameters for relative treatment effects.
  vector[n_treatments - 1] d_est;

  for (t in 2:n_treatments) d_est[t - 1] = theta[t] - theta[1];

}
```

Arm-based adjusted method *ABadj*

```
data{
  // IPD
  int <lower=1>  n_patients_ipd;
  int <lower=1>  study_ipd[n_patients_ipd];
  int <lower=1>  treatment_ipd[n_patients_ipd];
  int <lower=1>  baseline_ipd[n_patients_ipd];
  int <lower=1>  arm_ipd[n_patients_ipd];
  int <lower=0, upper=1>  index_ipd[n_patients_ipd];
  int <lower=0>  Y[n_patients_ipd];
  // AD
  int <lower=1>  n_arms_ad;
  int <lower=1>  study_ad[n_arms_ad];
  int <lower=1>  treatment_ad[n_arms_ad];
  int <lower=1>  baseline_ad[n_arms_ad];
  int <lower=0, upper=1>  index_ad[n_arms_ad];
  int <lower=1>  n[n_arms_ad];
  int <lower=0>  r[n_arms_ad];
  // Prior parameters
  real <lower=0>  prior_norm_sd;
  real <lower=0>  prior_unif_upper;
  //
  int <lower=2> n_studies_ipd;
  int nu_indices_ipd[n_studies_ipd, 2];
  // Covariate matrices
  int <lower=1> n_cov;
  vector[n_cov] X_ipd[n_patients_ipd];
  vector[n_cov] X_ad[n_arms_ad];
}

transformed data{
  int <lower=2> n_treatments = max(append_array(treatment_ipd, treatment_ad));
  int <lower=2> n_arms_ipd = max(arm_ipd);
  int <lower=2> n_studies_ad = max(study_ad);
}

parameters{
  row_vector[n_cov] beta;
  vector[n_arms_ipd + n_arms_ad] u_nu;
  vector[n_treatments] theta;
  real <lower=0> tau;
  real <lower=-1, upper=1> rho;
}


transformed parameters{
  matrix[2, 2] L;
```

```
  vector[n_arms_ipd + n_arms_ad] nu;
  vector[n_patients_ipd] lin_pred_ipd;
  vector[n_arms_ad] lin_pred_ad;

  //########################
  // CHOLESKY DECOMPOSITION
  //########################

  L[1, 1] = 1;     L[1, 2] = 0;
  L[2, 1] = rho;  L[2, 2] = sqrt(1 - rho ^ 2);

  //##########################
  // RANDOM TREATMENT EFFECTS
  //##########################

  for(stud in 1:n_studies_ipd){
    int s = nu_indices_ipd[stud, 1];
    int t = nu_indices_ipd[stud, 2];
    if (s == t) {                      // single-arm trials
      nu[s] = tau * u_nu[s];
    } else {                           // randomised trials
      nu[s:t] = tau * L * u_nu[s:t];
    }
  }

  for(stud in 1:n_studies_ad){
    int s = n_arms_ipd + (2 * stud - 1);
    nu[s:s + 1] = tau * L * u_nu[s:s + 1];
  }

  //###################
  // LINEAR PREDICTORS
  //###################

  for (patient in 1:n_patients_ipd){
    lin_pred_ipd[patient] = theta[treatment_ipd[patient]] +
      nu[arm_ipd[patient]] +
      beta * X_ipd[patient];
  }

  for (arm in 1:n_arms_ad){
    lin_pred_ad[arm] = theta[treatment_ad[arm]] +
      nu[n_arms_ipd + arm] +
      beta * X_ad[arm];
  }
}

model{
  // Implicit logit tranformation onto linear predictor scale.
```

```
  Y ~ bernoulli_logit(lin_pred_ipd);
  r ~ binomial_logit(n, lin_pred_ad);

  // Random variability for random effects.
  u_nu ~ std_normal();

  // Prior distributions
  theta ~ normal(0, prior_norm_sd);
  tau ~ uniform(0.0001, prior_unif_upper);
  rho ~ uniform(-0.999, 0.999);
  beta ~ normal(0, prior_norm_sd);
}

generated quantities{
  // Basic parameters for relative treatment effects.
  vector[n_treatments - 1] d_est;

  for (t in 2:n_treatments) d_est[t - 1] = theta[t] - theta[1];

}
```

Arm-based adjusted ecological bias method *ABadjEb*

```
data{
  // IPD
  int <lower=1>  n_patients_ipd;
  int <lower=1>  study_ipd[n_patients_ipd];
  int <lower=1>  treatment_ipd[n_patients_ipd];
  int <lower=1>  baseline_ipd[n_patients_ipd];
  int <lower=1>  arm_ipd[n_patients_ipd];
  int <lower=0, upper=1>  index_ipd[n_patients_ipd];
  int <lower=0>  Y[n_patients_ipd];
  // AD
  int <lower=1>  n_arms_ad;
  int <lower=1>  study_ad[n_arms_ad];
  int <lower=1>  treatment_ad[n_arms_ad];
  int <lower=1>  baseline_ad[n_arms_ad];
  int <lower=0, upper=1>  index_ad[n_arms_ad];
  int <lower=1>  n[n_arms_ad];
  int <lower=0>  r[n_arms_ad];
  // Prior parameters
  real <lower=0>  prior_norm_sd;
  real <lower=0>  prior_unif_upper;
  //
  int <lower=2> n_studies_ipd;
  int nu_indices_ipd[n_studies_ipd, 2];
  // Covariate matrices
  int <lower=1> n_cov;
  vector[n_cov] X_ipd[n_patients_ipd];
  vector[n_cov] X_ad[n_arms_ad];
  vector[n_cov] Xbar_ipd[n_patients_ipd];
}

transformed data{
  int <lower=2> n_treatments = max(append_array(treatment_ipd, treatment_ad));
  int <lower=2> n_arms_ipd = max(arm_ipd);
  int <lower=2> n_studies_ad = max(study_ad);
}

parameters{
  vector[n_arms_ipd + n_arms_ad] u_nu;
  vector[n_treatments] theta;
  real <lower=0> tau;
  real <lower=-1, upper=1> rho;
  row_vector[n_cov] beta_a;
  row_vector[n_cov] beta_w;
}
```

```
transformed parameters{
  matrix[2, 2] L;
  vector[n_arms_ipd + n_arms_ad] nu;
  vector[n_patients_ipd] lin_pred_ipd;
  vector[n_arms_ad] lin_pred_ad;

  //########################
  // CHOLESKY DECOMPOSITION
  //########################

  L[1, 1] = 1;     L[1, 2] = 0;
  L[2, 1] = rho;  L[2, 2] = sqrt(1 - rho ^ 2);

  //##########################
  // RANDOM TREATMENT EFFECTS
  //##########################

  for(stud in 1:n_studies_ipd){
    int s = nu_indices_ipd[stud, 1];
    int t = nu_indices_ipd[stud, 2];
    if (s == t) {                    // single-arm trials
      nu[s] = tau * u_nu[s];
    } else {                         // randomised trials
      nu[s:t] = tau * L * u_nu[s:t];
    }
  }

  for(stud in 1:n_studies_ad){
    int s = n_arms_ipd + (2 * stud - 1);
    nu[s:s + 1] = tau * L * u_nu[s:s + 1];
  }

  //###################
  // LINEAR PREDICTORS
  //###################

  for (patient in 1:n_patients_ipd){
    lin_pred_ipd[patient] = theta[treatment_ipd[patient]] +
      nu[arm_ipd[patient]] +
      beta_a * Xbar_ipd[patient] +
      beta_w * (X_ipd[patient] - Xbar_ipd[patient]);
  }

  for (arm in 1:n_arms_ad){
    lin_pred_ad[arm] = theta[treatment_ad[arm]] +
      nu[n_arms_ipd + arm] +
      beta_a * X_ad[arm];
  }
}
```

```
model{
  // Implicit logit tranformation onto linear predictor scale.
  Y ~ bernoulli_logit(lin_pred_ipd);
  r ~ binomial_logit(n, lin_pred_ad);

  // Random variability for random effects.
  u_nu ~ std_normal();

  // Prior distributions
  theta ~ normal(0, prior_norm_sd);
  tau ~ uniform(0.0001, prior_unif_upper);
  rho ~ uniform(-0.999, 0.999);
  beta_a ~ normal(0, prior_norm_sd);
  beta_w ~ normal(0, prior_norm_sd);
}

generated quantities{
  // Basic parameters for relative treatment effects.
  vector[n_treatments - 1] d_est;

  for (t in 2:n_treatments) d_est[t - 1] = theta[t] - theta[1];

}
```

Appendix D - MCMC diagnostic plots

Contrast-based unadjusted method with exchangeable baseline response (*CBunadjEx*)

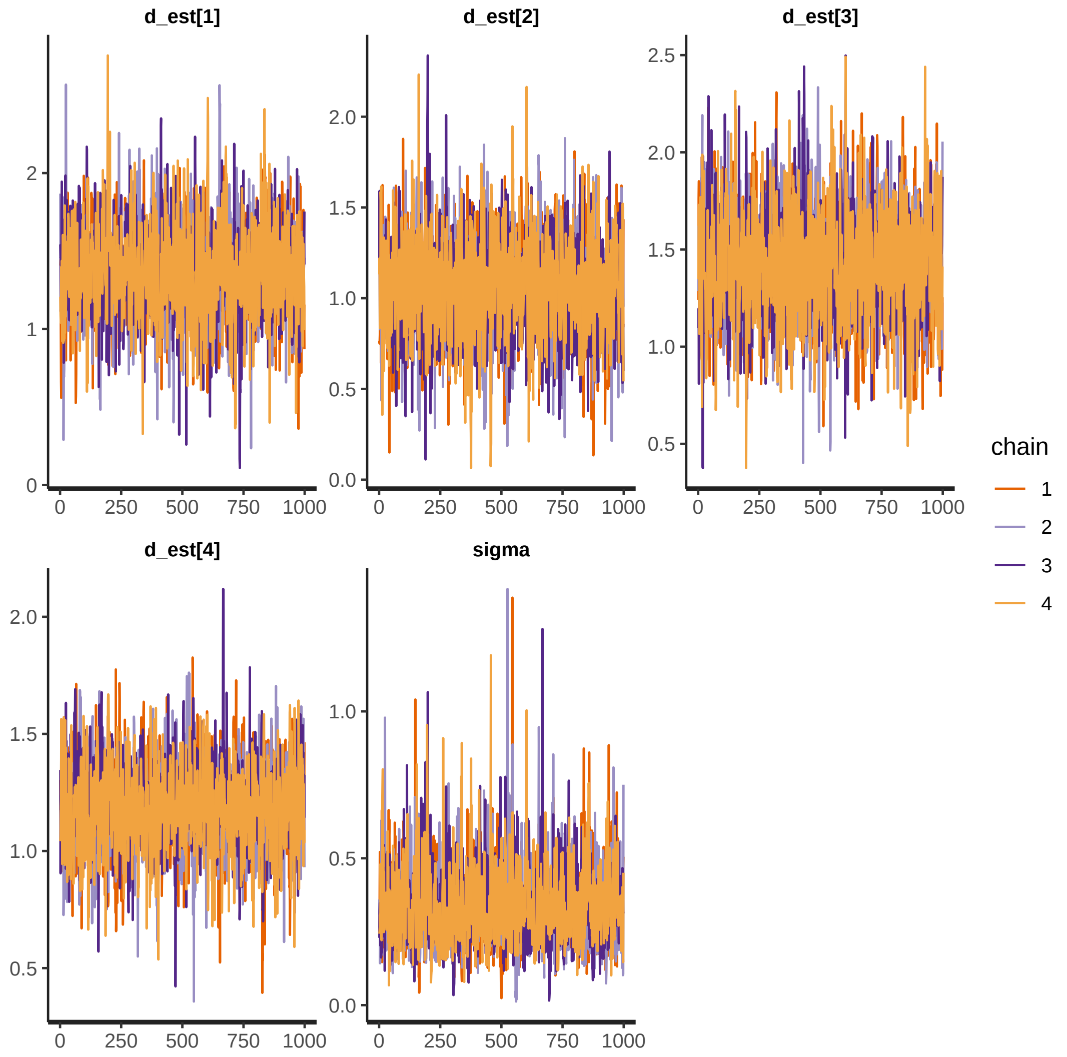

Figure D1: Trace plot

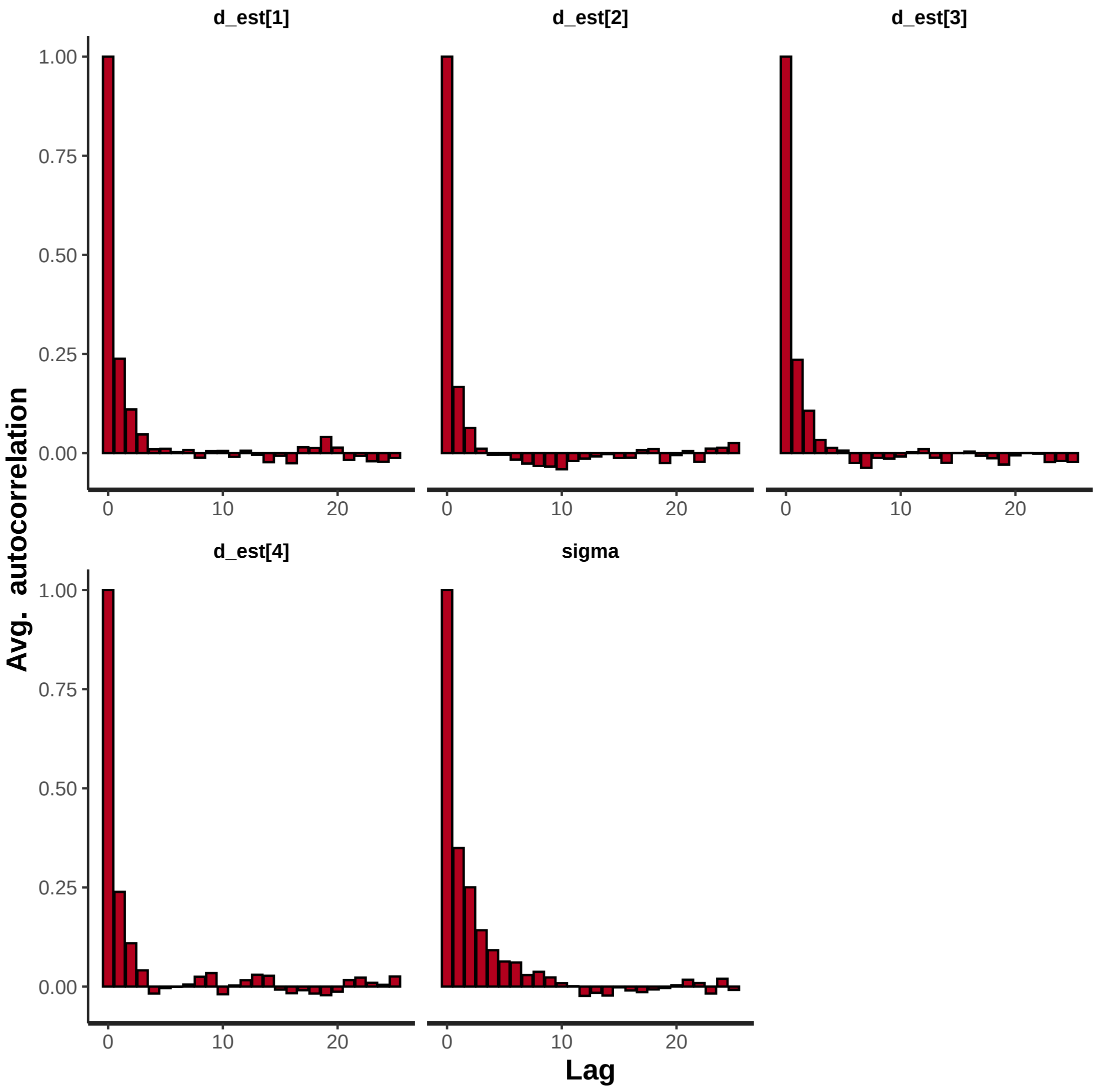

Figure D2: Autocorrelation plot

Contrast-based adjusted method with exchangeable baseline response (*CBadjEx*)

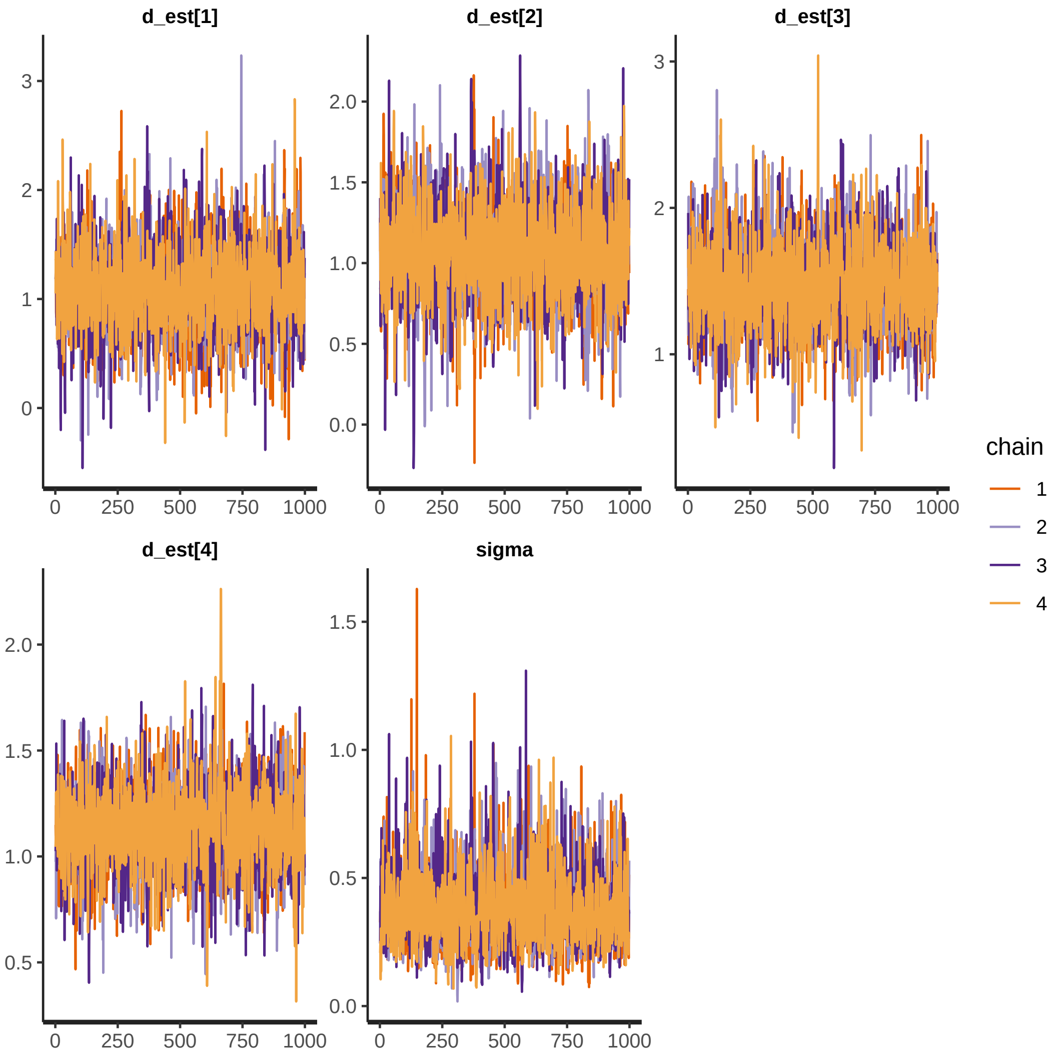

Figure D3: Trace plot

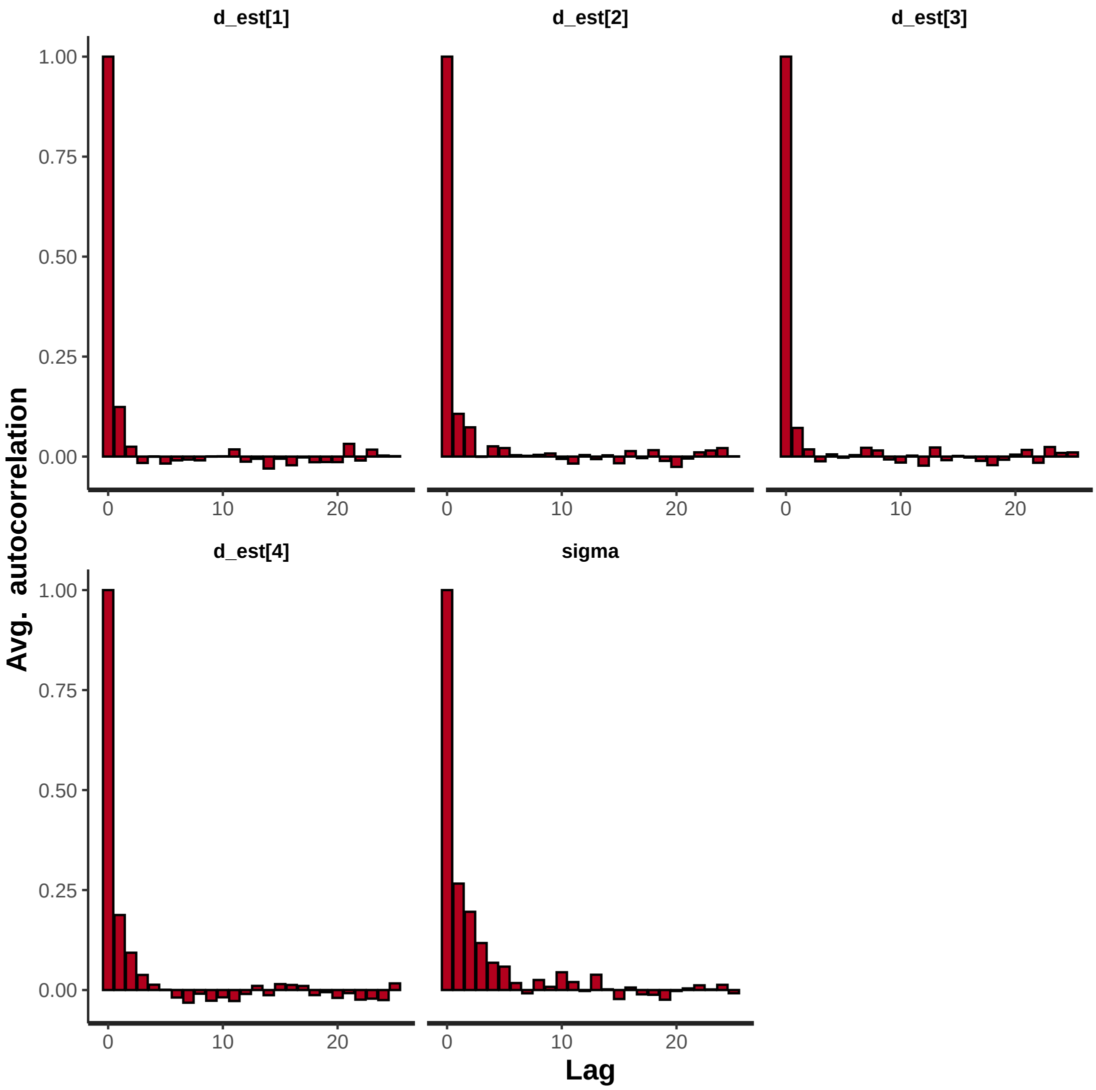

Figure D4: Autocorrelation plot

Arm-based adjusted method (*ABadj*)

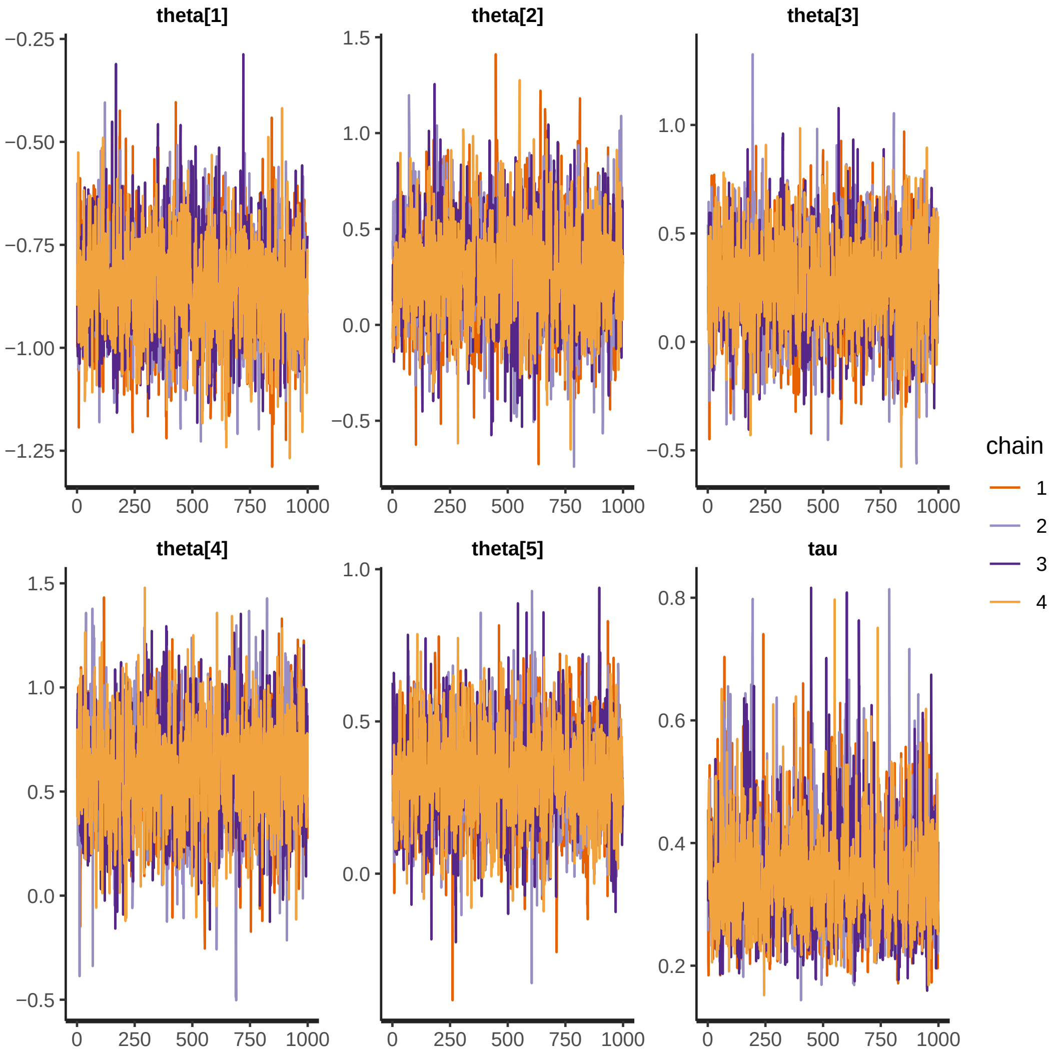

Figure D5: Trace plot

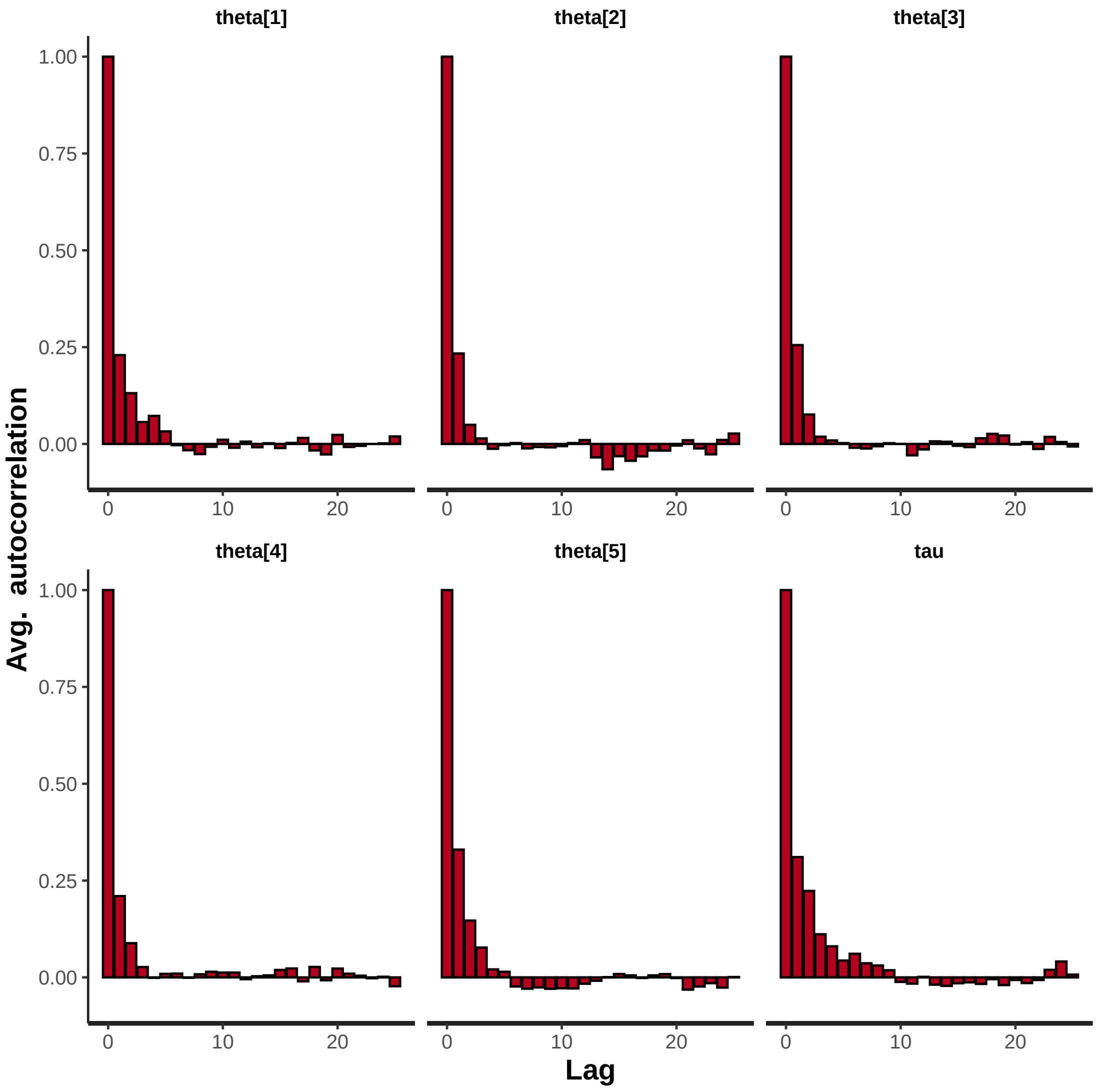

Figure D6: Autocorrelation plot

Appendix E - Sensitivity analysis on prior distribution for between-study heterogeneity parameters

Parameter estimates presented in the following tables are the result of a sensitivity analysis concerning the non-informative Uniform prior distribution placed-on between-study heterogeneity parameters. A $U(0,2)$ prior distribution was used in the original analysis, whilst a $U(0,10)$ has been used in this sensitivity analysis.

Parameter estimates for contrast-based unadjusted methods

Table E1: Median and 95% credible interval estimates for model parameters corresponding to the contrast-based unadjusted methods.

Parameter	CBunadjInd1	CBunadjEx1	CBunadjInd2	CBunadjEx2
ϕ_1	-1.06 (-1.37, -0.77)	-1.03 (-1.32, -0.76)	-0.90 (-1.50, -0.27)	-0.91 (-1.64, -0.24)
ϕ_2	-1.11 (-1.33, -0.90)	-1.08 (-1.30, -0.87)	-0.77 (-1.39, -0.16)	-0.76 (-1.45, -0.07)
ϕ_3	-1.11 (-1.58, -0.66)	-1.00 (-1.38, -0.67)	-1.09 (-1.57, -0.67)	-0.98 (-1.40, -0.63)
ϕ_4	-1.53 (-2.17, -0.99)	-1.22 (-1.72, -0.82)	-1.53 (-2.20, -0.97)	-1.19 (-1.71, -0.79)
ϕ_5	-0.90 (-1.21, -0.61)	-0.88 (-1.15, -0.62)	-0.91 (-1.21, -0.62)	-0.87 (-1.14, -0.60)
ϕ_6	-1.11 (-2.08, -0.18)	-0.96 (-1.52, -0.44)	-1.11 (-2.07, -0.19)	-0.92 (-1.55, -0.37)
ϕ_7	-0.59 (-1.18, -0.01)	-0.72 (-1.13, -0.28)	-0.60 (-1.17, -0.04)	-0.69 (-1.12, -0.25)
ϕ_8	-0.92 (-1.28, -0.58)	-0.91 (-1.22, -0.60)	-0.92 (-1.28, -0.58)	-0.88 (-1.21, -0.60)
ϕ_9	-1.18 (-1.55, -0.80)	-1.12 (-1.46, -0.81)	-1.18 (-1.55, -0.78)	-1.12 (-1.45, -0.80)
ϕ_{10}	-0.39 (-0.76, -0.03)	-0.52 (-0.85, -0.18)	-0.39 (-0.75, -0.02)	-0.51 (-0.82, -0.16)
ϕ_{11}	-0.11 (-0.43, 0.20)	-0.28 (-0.58, 0.03)	-0.11 (-0.42, 0.21)	-0.27 (-0.58, 0.05)
ϕ_{12}	-0.87 (-1.36, -0.41)	-0.89 (-1.24, -0.52)	-0.88 (-1.35, -0.42)	-0.87 (-1.24, -0.51)
ϕ_{13}	-0.59 (-1.03, -0.20)	-0.64 (-1.00, -0.29)	-0.59 (-1.01, -0.19)	-0.62 (-0.99, -0.27)
ϕ_{14}	-1.06 (-1.39, -0.76)	-1.01 (-1.31, -0.76)	-1.06 (-1.38, -0.76)	-1.01 (-1.29, -0.73)
d_1	0.00 (0.00, 0.00)	0.00 (0.00, 0.00)	0.00 (0.00, 0.00)	0.00 (0.00, 0.00)
d_2	1.37 (0.65, 2.10)	1.35 (0.76, 1.92)	1.12 (0.29, 1.94)	1.11 (0.32, 1.94)
d_3	1.05 (0.37, 1.69)	1.06 (0.56, 1.57)	1.04 (0.41, 1.70)	1.05 (0.54, 1.54)
d_4	1.59 (0.91, 2.28)	1.43 (0.88, 1.99)	1.57 (0.92, 2.26)	1.40 (0.85, 1.98)
d_5	1.14 (0.69, 1.57)	1.17 (0.82, 1.52)	1.13 (0.70, 1.58)	1.16 (0.82, 1.52)
σ	0.41 (0.19, 0.87)	0.32 (0.13, 0.68)	0.41 (0.18, 0.86)	0.32 (0.10, 0.67)

Parameter estimates for arm-based unadjusted methods

Table E2: Median and 95% credible interval estimates for model parameters corresponding to the contrast-based unadjusted and adjusted methods.

Parameter	CBunadjEx2	CBadjEx2	CBadjEbEx2
m_ϕ	-0.83 (-1.10, -0.57)	-0.84 (-1.07, -0.61)	-0.84 (-1.07, -0.62)
σ_ϕ	0.34 (0.18, 0.62)	0.27 (0.12, 0.55)	0.27 (0.13, 0.55)
ϕ_1	-0.91 (-1.64, -0.24)	-0.93 (-1.56, -0.38)	-0.93 (-1.57, -0.36)
ϕ_2	-0.76 (-1.45, -0.07)	-0.74 (-1.33, -0.14)	-0.74 (-1.34, -0.11)
ϕ_3	-0.98 (-1.40, -0.63)	-0.94 (-1.31, -0.60)	-0.94 (-1.32, -0.61)
ϕ_4	-1.19 (-1.71, -0.79)	-0.95 (-1.43, -0.54)	-0.95 (-1.48, -0.54)
ϕ_5	-0.87 (-1.14, -0.60)	-0.66 (-0.99, -0.28)	-0.66 (-0.98, -0.26)
ϕ_6	-0.92 (-1.55, -0.37)	-0.93 (-1.49, -0.43)	-0.93 (-1.48, -0.47)
ϕ_7	-0.69 (-1.12, -0.25)	-0.77 (-1.17, -0.36)	-0.77 (-1.18, -0.34)
ϕ_8	-0.88 (-1.21, -0.60)	-1.01 (-1.38, -0.69)	-1.01 (-1.39, -0.70)
ϕ_9	-1.12 (-1.45, -0.80)	-1.06 (-1.39, -0.76)	-1.05 (-1.39, -0.75)
ϕ_{10}	-0.51 (-0.82, -0.16)	-0.55 (-0.86, -0.22)	-0.55 (-0.86, -0.20)
ϕ_{11}	-0.27 (-0.58, 0.05)	-0.66 (-1.09, -0.18)	-0.66 (-1.10, -0.20)
ϕ_{12}	-0.87 (-1.24, -0.51)	-0.83 (-1.19, -0.46)	-0.82 (-1.19, -0.46)
ϕ_{13}	-0.62 (-0.99, -0.27)	-0.63 (-0.97, -0.26)	-0.63 (-0.97, -0.27)
ϕ_{14}	-1.01 (-1.29, -0.73)	-1.06 (-1.36, -0.78)	-1.07 (-1.38, -0.79)
d_1	0.00 (0.00, 0.00)	0.00 (0.00, 0.00)	0.00 (0.00, 0.00)
d_2	1.11 (0.32, 1.94)	1.09 (0.36, 1.81)	1.10 (0.34, 1.85)
d_3	1.05 (0.54, 1.54)	1.07 (0.51, 1.58)	1.07 (0.51, 1.61)
d_4	1.40 (0.85, 1.98)	1.49 (0.92, 2.08)	1.48 (0.89, 2.05)
d_5	1.16 (0.82, 1.52)	1.13 (0.76, 1.51)	1.13 (0.75, 1.54)
σ	0.32 (0.10, 0.67)	0.36 (0.16, 0.71)	0.36 (0.17, 0.75)
α^a		-0.10 (-0.20, 0.00)	-0.10 (-0.20, 0.00)
α^w			0.00 (-0.02, 0.01)

Parameter estimates for contrast-based methods applied to the artificial dataset

Table E3: Median and 95% credible interval estimates for model parameters corresponding to the arm-based unadjusted models.

Parameter	ABunadj1	ABunadj2
θ_1	-0.88 (-1.10, -0.67)	-0.83 (-1.09, -0.58)
θ_2	0.33 (-0.17, 0.80)	0.29 (-0.25, 0.82)
θ_3	0.24 (-0.22, 0.66)	0.24 (-0.20, 0.67)
θ_4	0.44 (0.00, 0.90)	0.45 (-0.02, 0.91)
θ_5	0.36 (0.06, 0.66)	0.38 (0.06, 0.71)
ν_1	-0.12 (-0.48, 0.21)	-0.16 (-0.68, 0.38)
ν_2	-0.19 (-0.66, 0.30)	0.14 (-0.38, 0.68)
ν_3	0.10 (-0.38, 0.60)	0.04 (-0.40, 0.50)
ν_4	-0.21 (-0.50, 0.08)	-0.22 (-0.68, 0.16)
ν_5	0.04 (-0.39, 0.50)	-0.05 (-0.53, 0.45)
ν_6	-0.20 (-0.62, 0.21)	-0.46 (-1.00, 0.01)
ν_7	-0.04 (-0.52, 0.41)	0.04 (-0.42, 0.54)
ν_8	-0.41 (-0.96, 0.02)	-0.04 (-0.38, 0.31)
ν_9	0.05 (-0.43, 0.50)	-0.13 (-0.68, 0.40)
ν_{10}	0.01 (-0.33, 0.34)	-0.02 (-0.61, 0.65)
ν_{11}	-0.12 (-0.65, 0.40)	0.07 (-0.32, 0.47)
ν_{12}	0.00 (-0.61, 0.58)	0.14 (-0.34, 0.68)
ν_{13}	0.08 (-0.31, 0.47)	0.00 (-0.37, 0.37)
ν_{14}	0.17 (-0.31, 0.68)	-0.09 (-0.48, 0.30)
ν_{15}	0.01 (-0.36, 0.39)	-0.55 (-0.94, -0.20)
ν_{16}	-0.05 (-0.41, 0.30)	-0.24 (-0.65, 0.16)
ν_{17}	-0.54 (-0.92, -0.18)	0.14 (-0.33, 0.60)
ν_{18}	-0.20 (-0.59, 0.18)	0.37 (0.00, 0.77)
ν_{19}	0.14 (-0.33, 0.63)	0.12 (-0.26, 0.47)
ν_{20}	0.40 (0.04, 0.78)	0.64 (0.28, 1.02)
ν_{21}	0.12 (-0.23, 0.49)	-0.14 (-0.65, 0.33)
ν_{22}	0.67 (0.34, 1.05)	-0.02 (-0.46, 0.42)
ν_{23}	-0.13 (-0.63, 0.36)	0.47 (0.10, 0.89)
ν_{24}	0.02 (-0.39, 0.45)	0.13 (-0.29, 0.56)
ν_{25}	0.48 (0.12, 0.88)	-0.04 (-0.38, 0.30)
ν_{26}	0.17 (-0.25, 0.58)	-0.21 (-0.57, 0.14)
ν_{27}	-0.02 (-0.36, 0.31)	
ν_{28}	-0.17 (-0.51, 0.16)	
τ	0.33 (0.22, 0.52)	0.35 (0.23, 0.55)
ρ	0.28 (-0.40, 0.81)	0.31 (-0.41, 0.82)

Parameter estimates for arm-based methods applied to the artificial dataset

Table E4: Median and 95% credible interval estimates for model parameters corresponding to the arm-based unadjusted and adjusted models.

Parameter	ABunadj2	ABadj2	ABadjEb2
θ_1	-0.83 (-1.09, -0.58)	-0.85 (-1.09, -0.60)	-0.85 (-1.11, -0.61)
θ_2	0.29 (-0.25, 0.82)	0.26 (-0.26, 0.77)	0.27 (-0.27, 0.77)
θ_3	0.24 (-0.20, 0.67)	0.28 (-0.13, 0.68)	0.27 (-0.18, 0.71)
θ_4	0.45 (-0.02, 0.91)	0.60 (0.11, 1.07)	0.60 (0.10, 1.07)
θ_5	0.38 (0.06, 0.71)	0.32 (0.02, 0.62)	0.32 (0.00, 0.63)
ν_1	-0.16 (-0.68, 0.38)	-0.21 (-0.74, 0.30)	-0.22 (-0.75, 0.31)
ν_2	0.14 (-0.38, 0.68)	0.22 (-0.30, 0.75)	0.21 (-0.28, 0.75)
ν_3	0.04 (-0.40, 0.50)	0.04 (-0.38, 0.45)	0.05 (-0.40, 0.49)
ν_4	-0.22 (-0.68, 0.16)	-0.18 (-0.61, 0.22)	-0.17 (-0.61, 0.22)
ν_5	-0.05 (-0.53, 0.45)	0.09 (-0.40, 0.62)	0.09 (-0.39, 0.61)
ν_6	-0.46 (-1.00, 0.01)	-0.31 (-0.86, 0.16)	-0.31 (-0.89, 0.16)
ν_7	0.04 (-0.42, 0.54)	0.08 (-0.34, 0.53)	0.08 (-0.35, 0.57)
ν_8	-0.04 (-0.38, 0.31)	0.12 (-0.26, 0.56)	0.13 (-0.26, 0.54)
ν_9	-0.13 (-0.68, 0.40)	-0.24 (-0.83, 0.29)	-0.24 (-0.83, 0.29)
ν_{10}	-0.02 (-0.61, 0.65)	-0.01 (-0.63, 0.61)	-0.01 (-0.62, 0.63)
ν_{11}	0.07 (-0.32, 0.47)	0.11 (-0.28, 0.50)	0.12 (-0.27, 0.52)
ν_{12}	0.14 (-0.34, 0.68)	0.09 (-0.41, 0.57)	0.08 (-0.40, 0.58)
ν_{13}	0.00 (-0.37, 0.37)	-0.06 (-0.46, 0.30)	-0.07 (-0.48, 0.32)
ν_{14}	-0.09 (-0.48, 0.30)	-0.14 (-0.52, 0.24)	-0.14 (-0.54, 0.24)
ν_{15}	-0.55 (-0.94, -0.20)	-0.45 (-0.84, -0.09)	-0.45 (-0.85, -0.07)
ν_{16}	-0.24 (-0.65, 0.16)	-0.24 (-0.63, 0.13)	-0.23 (-0.62, 0.16)
ν_{17}	0.14 (-0.33, 0.60)	0.07 (-0.37, 0.51)	0.08 (-0.42, 0.54)
ν_{18}	0.37 (0.00, 0.77)	0.39 (0.01, 0.79)	0.39 (0.02, 0.82)
ν_{19}	0.12 (-0.26, 0.47)	-0.09 (-0.54, 0.35)	-0.09 (-0.54, 0.36)
ν_{20}	0.64 (0.28, 1.02)	0.38 (-0.08, 0.88)	0.38 (-0.09, 0.88)
ν_{21}	-0.14 (-0.65, 0.33)	-0.09 (-0.57, 0.38)	-0.07 (-0.57, 0.42)
ν_{22}	-0.02 (-0.46, 0.42)	-0.01 (-0.45, 0.41)	-0.01 (-0.43, 0.42)
ν_{23}	0.47 (0.10, 0.89)	0.53 (0.18, 0.94)	0.54 (0.16, 0.98)
ν_{24}	0.13 (-0.29, 0.56)	0.16 (-0.27, 0.59)	0.17 (-0.25, 0.62)
ν_{25}	-0.04 (-0.38, 0.30)	-0.04 (-0.38, 0.28)	-0.05 (-0.38, 0.30)
ν_{26}	-0.21 (-0.57, 0.14)	-0.24 (-0.61, 0.09)	-0.24 (-0.61, 0.10)
τ	0.35 (0.23, 0.55)	0.33 (0.21, 0.53)	0.33 (0.21, 0.55)
ρ	0.31 (-0.41, 0.82)	0.22 (-0.51, 0.80)	0.24 (-0.54, 0.81)
β^a		-0.06 (-0.15, 0.02)	-0.06 (-0.15, 0.02)
β^w			0.00 (-0.02, 0.01)
